# Bioenergetic trophic trade‐offs determine mass‐dependent extinction thresholds across the Cenozoic

**DOI:** 10.1002/ecy.70390

**Published:** 2026-05-04

**Authors:** Justin D. Yeakel, Matthew C. Hutchinson, Christopher P. Kempes, Paul L. Koch, Pedro D. S. Ugarte, Jacquelyn L. Gill, Mathias M. Pires

**Affiliations:** ^1^ Life & Environmental Sciences, UC Merced Merced California USA; ^2^ The Santa Fe Institute Santa Fe New Mexico USA; ^3^ Earth & Planetary Sciences, UC Santa Cruz Santa Cruz California USA; ^4^ Programa de Pós‐Graduação em Ecologia Instituto de Biologia, Universidade Estadual de Campinas Campinas Brazil; ^5^ School of Biology and Ecology Climate Change Institute University of Maine Derry Maine USA; ^6^ Departamento de Biologia Animal Instituto de Biologia, Universidade Estadual de Campinas Campinas Brazil

**Keywords:** body mass, macroevolution, megafauna, predation, trophic interactions

## Abstract

Body size constrains trophic interactions, shaping the feasibility of species' populations. Over macroevolutionary timescales, these constraints feed back to shape selection on body size and diet. We develop a bioenergetic, three‐level trophic framework—typical of terrestrial mammalian ecosystems—to explore how bioenergetic trade‐offs emerging from predator–prey interactions constrain coexistence. We show that interactions among predators, prey, and subsidies destabilize populations at both small and large sizes, matching observed limits to predator size and diet. These instabilities constrain coexistence and highlight a feasible predator size range of ca. 40–110 kg, spanning the mean size of terrestrial Cenozoic hypercarnivores. Finally, we show that decreased dietary selectivity confers a fitness advantage to larger carnivores that wanes at the largest sizes, aligning with diet estimates for contemporary and Pleistocene species. Our results underscore that ecological pressures emerging from trophic interactions, rooted in energetics, give rise to selective forces driving observed macroevolutionary patterns.

## INTRODUCTION

Terrestrial mammalian carnivores span a wide range of body sizes, from smaller bodied mustelids with generalist diets, including vertebrates, invertebrates, and plants, to larger bodied vertebrate specialists such as wolves (*Canis lupus*) and lions (*Panthera leo*). Body size drives the energetic demands of organisms, governing changes in resource assimilation (Bhat et al., 2020; Hou et al., 2008) and energetic allocation to somatic growth, maintenance, and reproduction (Kempes et al., 2012; West et al., 1997) across individual lifetimes (Lindstedt & Schaeffer, 2002). Whereas organismal metabolic rates are central to determining which resources are energetically rewarding and how much an organism must eat to meet its energetic demand (Carbone et al., 1999; Hou et al., 2008), body size also imposes biomechanical constraints on the size of prey that can be subjugated and consumed (Sorkin, 2008). The viability of a predator population is thus determined by the balance between a species' energetic demands and the ability of its prey to sustain predation while avoiding collapse (Carbone et al., 2007; Otto et al., 2007).

Whereas the central role of body size in structuring ecological systems is clear, how species interactions may have contributed to size evolution throughout the Cenozoic is less so. Long after the rapid expansion of mammalian body size classes following the K‐Pg extinction event 66 million years ago (Alroy, 1998; Clauset & Erwin, 2008; Smith & Lyons, 2011), the cooler, drier, and increasingly open habitat of a post‐Eocene world (Andermann et al., 2022; Strömberg, 2011) coincided with the appearance of maximal body sizes among herbivorous (at ca. 17400 kg) and carnivorous (at ca. 1000 kg) mammalian lineages across multiple continents (Smith et al., 2010). With the evolution of increasingly large mammals came bioenergetic constraints (Weitz & Levin, 2006) that shape whether the coexistence of a given set of species is feasible and thus can, in principle, coexist (Saavedra, 2024). These bioenergetic boundaries may thus be explicitly connected to population instabilities that arise from trophic interactions, where consumption by the predator population may suppress its prey to the point of collapse—a function of the body sizes and associated population densities of each (Rallings et al., 2024; Yeakel et al., 2018). A theoretical framework that unites the bioenergetic constraints emerging from mammalian energetic trade‐offs with the dynamics of interacting species should provide important insight into the selective drivers shaping mammalian body size distributions—both past and present.

To understand how the bioenergetic trade‐offs associated with growth and mortality among predators and prey may impact the feasibility of species interactions and ultimately long‐term coexistence, we construct a generalizable three‐level trophic (i.e., tri‐trophic) dynamic model premised on the scaling of energetic demands with body size. By incorporating fundamental energetic trade‐offs in the vital rates governing animal growth and mortality, we aim to reconstruct potential ecological drivers influencing the evolution of body size.

**FIGURE 1:**
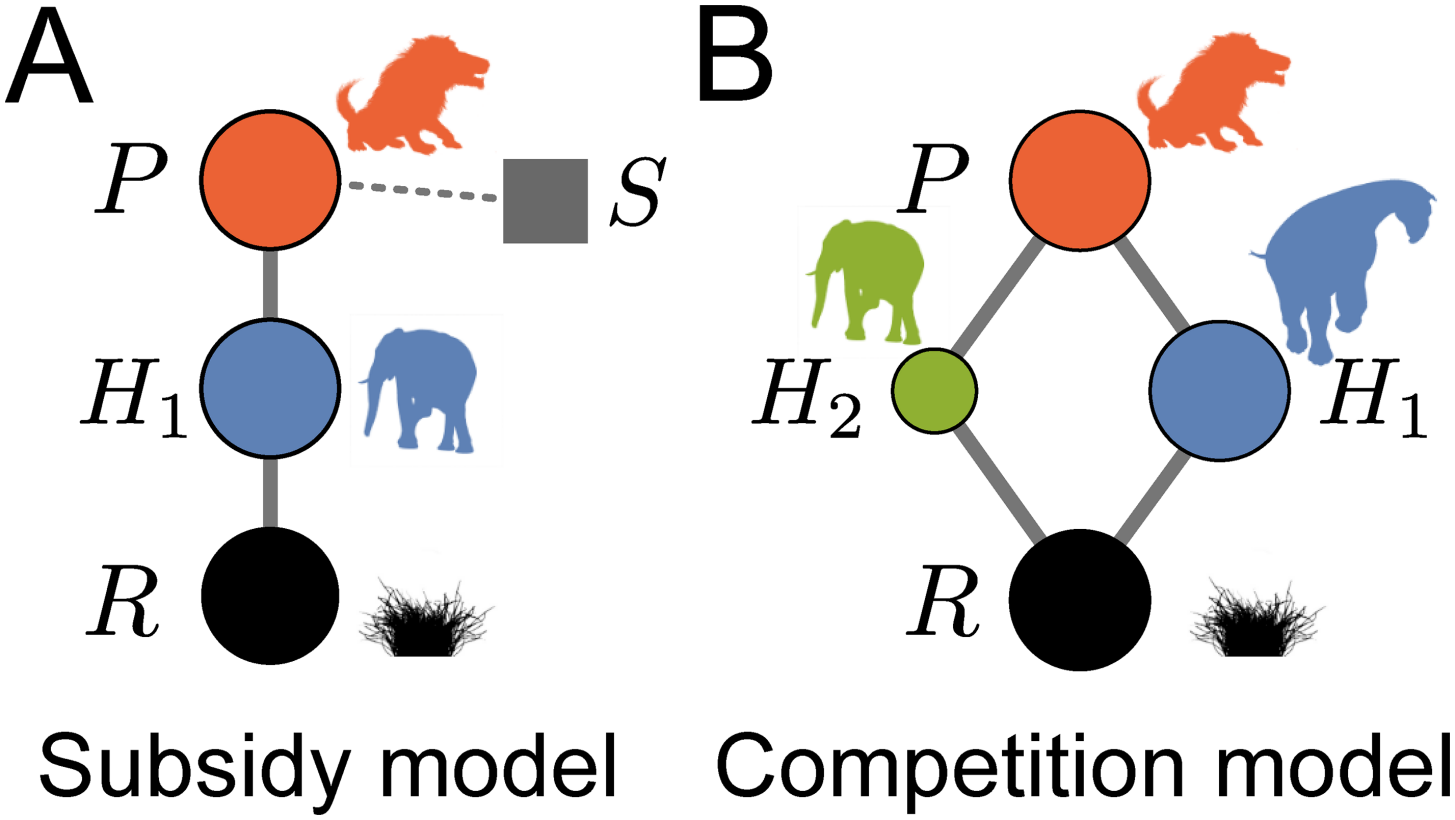
(A) The subsidy model, consisting of a predator population P with mass $M_{P}$ (red), an herbivore prey population $H_{1}$ with mass $E\left\{M_{H_{1}}|M_{P}\right\}$ (blue), an external subsidy S (gray), and a primary resource R (black). (B) The competition model, with the same elements as A., with the primary herbivore prey $H_{1}$ having mass $E\left\{M_{H_{1}}|M_{P}\right\}$, and the secondary herbivore prey $H_{2}$ with mass $\phi M_{H_{1}}$ (green). Silhouettes created by Justin D. Yeakel.

We analyze two tri‐trophic structures, or motifs, to investigate how predator and prey body sizes introduce bioenergetic constraints on species interactions. First, the subsidy model (one resource, one herbivore, and one predator with an external subsidy) represents the minimal system to isolate predator–prey negative feedback and then dial it down exogenously by adjusting predator reliance on resource subsidies (Figure 1A). This permits a clear evaluation of mass‐dependent feasibility/stability thresholds as the predator–herbivore coupling is relaxed by increasing the predator's subsidization. We note that this subsidy represents any alternative resource that does not respond dynamically to predator consumption. Second, the competition model (one resource, two herbivores, and one predator without a subsidy) assumes that top‐down pressure is redistributed across alternative prey populations (Figure 1B). Together these cases bracket common ecological contexts (external inputs versus multi‐prey foraging) without additional complexity, enabling us to explore how size‐structured interactions may shape feasibility, coexistence, and potential size thresholds under two mechanistically distinct ways of distributing the dynamics of trophic interactions.

We use these two tri‐trophic motifs to investigate and identify different energetic boundaries that determine the likelihood of population collapse as potential drivers of size selection. With the subsidy model, we aim to understand how increasing or decreasing the dynamic feedback between predator and prey (via tuning the predator's reliance on the subsidy) imposes size‐specific energetic limitations on the trophic interaction. With the competition model, we aim to understand how the coupled feedback between the predator and competing herbivores alters or adds to these size‐specific constraints. Together, this allows us to assess whether and to what extent body size impacts the potential for coexistence of some or all mammalian species, as well as the constraints these dynamics place on predator dietary breadth. We show that species at different trophic levels are exposed to distinct size‐specific risks, potentially shaping the structure and function of mammalian communities across the Cenozoic.

## MATERIALS AND METHODS

Our primary aim is to model the flow and storage of biomass between populations of interacting mammalian species as a function of body size. We then examine how energetic limitations constrain the feasibility of interacting populations, where feasibility is defined by those species retaining strictly positive densities at steady state. Using mechanistic models of metabolic trade‐offs governing mammalian ontogenetic growth, reproduction, and mortality, combined with allometric relationships capturing body size constraints on physiological and ecological traits, we describe a flexible tri‐trophic framework premised on the body sizes of each species. We then describe how the interactions between predators and herbivore prey are also body size‐dependent, and how empirical observations are used to condition interactions between the predator and its primary prey. We present a detailed description of the model and allometric derivations of the functions describing mammalian vital rates in Appendix S1: Section S1. See the archived Zenodo repository (Yeakel et al., 2026) to access the supplementary electronic data and code.

**FIGURE 2:**
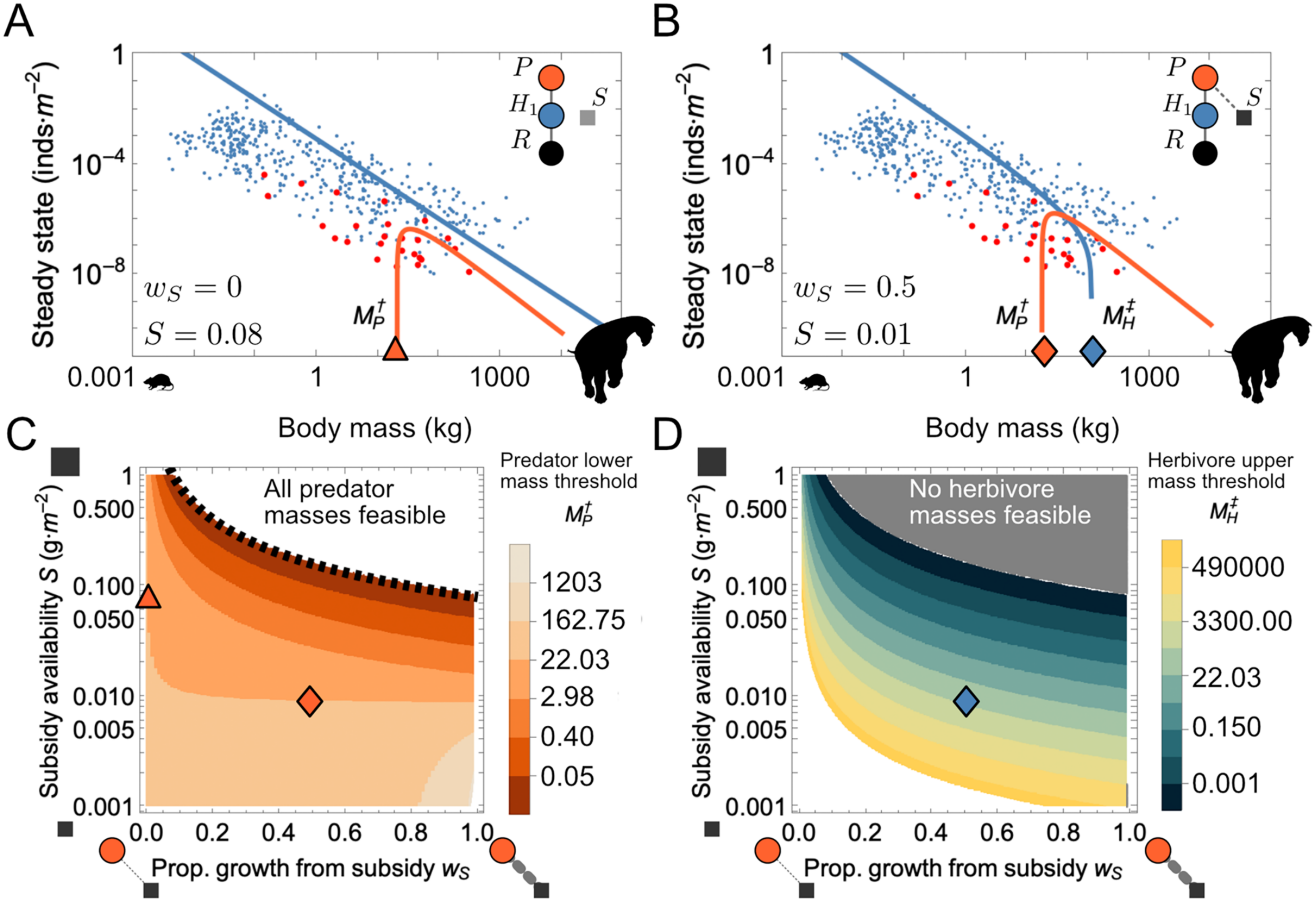
(A, B) Population densities of the herbivore and predator in the subsidy model. Blue and red lines denote the stable internal fixed points of the herbivore and predator populations, respectively, assuming (A) no reliance by the predator on a subsidy ($w_{S}=0$, S=0.08), and (B) with partial reliance by the predator on a subsidy ($w_{S}=0.5$, S=0.01). Blue and red points denote empirical herbivore and carnivore densities, respectively (Carbone & Gittleman, 2002, Damuth, 1987). A low‐mass threshold for the predator ($M_{P}^{†}$) is denoted by a red triangle symbol in (A); a low‐mass threshold for the predator ($M_{P}^{†}$), and a high‐mass threshold for the herbivore ($M_{H}^{‡}$) are denoted by the red and blue diamond symbols, respectively, in (B). (C) Predator low‐mass thresholds $M_{P}^{†}$, and (D) herbivore high‐mass thresholds $M_{H}^{‡}$ as a function of the percent of predator growth driven by consumption of the subsidy $w_{S}$, and subsidy availability S. The black dotted line in (C) represents the critical value of subsidization $S_{c}$ enabling predator masses ≥0.01 kg, and the white region denotes where all predator size classes are feasible, reliant entirely on subsidies. The gray region in (D) denotes where no herbivore size classes are feasible in the presence of a highly subsidized predator population. The triangle/diamond symbols in (A, B) are mapped to their corresponding locations in (C, D). Rodent silhouette obtained from www.publicdomainpictures.net (CC0 Public Domain); all others created by Justin D. Yeakel.

Here we describe a tri‐trophic food chain, consisting of a primary producer, one or more herbivore prey, and a predator. The energy available for somatic growth, maintenance, and reproduction of the predator population P is limited by its consumption of n herbivore prey populations $H_{i}$, for i=1,…,n. Each prey population is supported by the consumption of a single plant resource R. The growth of the predator population is fueled by the mortality it inflicts on its prey proportional to $w_{i}$, where $w_{i}$ is the proportional reliance of the predator on herbivore i. When the predator does not rely completely on its herbivore prey (i.e., $\sum_{i}w_{i}<1$), the remainder of predator growth is obtained from a constant subsidy ($w_{S}$) with availability S, which represents any other alternative prey or resource. The parameter w, as applied to subsidies, $w_{S}$, or herbivore prey, $w_{i}$, thus describes dietary selectivity across these potential foods, where a value w≈1 implies high selectivity on a specified resource, whereas w<1 implies low selectivity across multiple resources. Below we will consider two scenarios with this general framework: (1) the subsidy model, where the predator consumes a single herbivore prey $\left(n=1\right)$ and is otherwise supported by an external subsidy $\left(S>0\right)$ (Figure 1A); (2) the competition model, where the predator is supported by two herbivore prey species $\left(n=2\right)$ consuming the single resource R, without the aid of an external subsidy (S=0) (Figure 1B).

Whether two species engage in a trophic interaction given their respective traits drives the structures of food webs. The relationship that determines how the herbivore's mass $M_{H}$ relates to the predator's mass $M_{P}$ is provided by $E\left\{M_{H}|M_{P}\right\}$, where the function $E\left\{\cdot \right\}$ denotes the expectation, which we obtain from observational data. $E\left\{M_{H}|M_{P}\right\}$ follows a power law across $M_{P}$, where the slope has been shown to be roughly linear (Carbone et al., 1999) or slightly super‐linear (Uiterwaal et al., 2022), with theoretical expectations from resource use optimization predicting a slope of ca. 1.23 (DeLong, 2020). By integrating high‐resolution datasets for large‐bodied mammalian carnivores (Hayward, 2006; Hayward, Henschel, et al., 2006; Hayward, Hofmeyr, et al., 2006; Hayward & Kerley, 2005, 2008; Hayward, O'Brien, et al., 2006), this slope increases slightly to ca. 1.46 (see Appendix S1: Section S2). Throughout, we use the prefix “mega” to refer to species with body sizes ≥600 kg (Hayward & Kerley, 2005).

We assess ecological feasibility and coexistence across body mass combinations by evaluating whether the system has at least one steady state with strictly positive densities for some or all species' populations (R, $H_{i}$, P). A species is feasible when its steady state density is positive, and we define coexistence as feasibility (for the focal subset) plus local dynamical stability. In this case, a stable fixed point refers to a non‐zero steady state at which small perturbations in population densities decay back to that steady state. Accordingly, across predator masses (and by extension expected primary prey body mass, and across different secondary prey body masses given by ϕ), we classify species‐specific outcomes as infeasible (no positive internal steady state for a given species), or feasible and stable (coexistence for some or all species). Results are summarized as feasibility/stability maps over body mass space and as the fraction of feasible combinations within empirically observed mass ranges. Table 1 provides a summary of the parameters, their descriptions, and their value/units.

**TABLE 1:** List of parameters and their meanings.

Parameter	Description	Value/units
P	Predator density	g/m 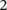
S	Constant subsidy density	g/m 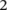
$H_{1}$	Primary herbivore prey density	g/m 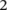
$H_{2}$	Secondary herbivore prey density	g/m 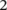
R	Plant resource density	g/m 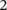
$w_{S}$	Predator reliance on subsidy	0:1
$w_{1}$	Predator reliance on primary prey	0:1
$M_{P}$	Predator body mass	kg
$M_{H_{1}}$	Primary herbivore prey body mass	$E\left\{M_{H}|M_{P}\right\}$ kg
$M_{H_{2}}$	Secondary herbivore prey body mass	$\phi M_{H_{1}}$ kg
ϕ	Size scaling of the secondary prey	0.1:2.0

*Note*: For a full model description and derivations of mass‐specific functions, see Appendix S1: Section S1.

## RESULTS

### Subsidized food chains and population thresholds

We observe that the subsidy model (Figure 1A), where the proportion of subsidies $w_{S}$ and the single herbivore prey $w_{1}$ to the diet of the predator sum to unity, has seven fixed points, but only one stable internal steady state for each particular combination of predator and herbivore body masses (Figure 2). This means that for each pair of predator and prey of a given size there is a single feasible steady state. When predators and prey are larger bodied, this steady state allows for the coexistence of both. For smaller predators and without subsidization, the steady state includes only the prey population (Figure 2A), while the coexistence of both requires that small‐bodied predators have access to resource subsidies ($w_{S}>0$). Despite the emergence of instabilities occurring at specific predator and herbivore body sizes (see below), the stable steady state densities of both fall within the range of empirical mammalian densities (Figure 2A,B), in line with similar investigations of mammalian dynamics that include mechanistic models of energetic trade‐offs (Bhat et al., 2020; Rallings et al., 2024; Yeakel et al., 2018). We note that while the steady state solutions for the predator ($P^{*}$) and herbivore prey ($H^{*}$) can be analytically expressed, they cannot be written in a compact form (see Supplemental Electronic Data in Yeakel et al. (2026)).

We observe specific limitations to the stability of the internal steady states for both the herbivore and predator as a function of their respective body mass and the dependence of the predator on external subsidization. We define the transition from stable to unstable dynamics as the body mass where a population declines to zero and describe that point as a ‘body mass threshold’. These thresholds are defined by transcritical bifurcations, a feature observed in other allometric models of population dynamics—typically at high‐mass limits (Rallings et al., 2024; Weitz & Levin, 2006). Throughout, we specify two distinct mass thresholds: a lower threshold (denoted by †), below which populations are infeasible, and an upper threshold (denoted by ‡) above which populations are infeasible.

When the predator specializes on its primary prey, we find that there is a lower limit to predator body mass, $M_{P}^{†}$, below which the population is infeasible such that its steady state vanishes (Figure 2A). Here, the smaller predator's reproductive output is stymied by its reliance on yet smaller prey, making the predator population unable to compensate for mortality unless externally subsidized. We note that the size threshold for predators in Figure 2A does not capture the lower bound of observed predator sizes but aligns with the lower bound for hypercarnivores. Most smaller sized carnivores represented in the figure are omnivores, relying to a large extent on non‐vertebrate resources (Carbone et al., 1999).

Our analysis shows that there is a level of subsidization where the lower mass threshold $M_{P}^{†}$ is minimized, thus enabling the existence of very small predators. This critical value of subsidy density $S=S_{c}$ enabling predator masses of 0.01 kg can be approximated as $S_{c}\approx S^{\mathrm{int}}w_{S}^{-1}$ where the intercept $S^{\mathrm{int}}=0.08$ g/m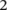 represents the subsidy required for a 0.01 kg predator obtaining all of its growth from the subsidy to achieve positive population densities (dotted line in Figure 2C). The subsidy density required to enable feasible populations at this lower size limit becomes greater as the predator's reliance on subsidies ($w_{S}$) decreases. While extreme subsidization can decrease the small‐mass threshold $M_{P}^{†}$ such that effectively all predator body sizes are feasible, increased specialization on mammalian prey (lower $w_{S}$) tends to raise $M_{P}^{†}$ (Figure 2C). For predators primarily reliant on herbivore prey, the small‐mass threshold thus establishes a lower bound on predator body size. We can estimate the average minimum expected viable body size for a predator specializing on mammalian prey, averaged across values of $w_{S}<0.1$, as $\left\langle M_{P}^{†}\right\rangle =15$ kg, though the primary mode falls closer to $M_{P}^{†}=22$ kg (Appendix S1: Section S3).

Increased subsidization leads to the emergence of a separate feasibility threshold occurring at the upper size limit of the herbivore prey population $M_{H}^{‡}$, representing its maximum feasible body size (Figure 2B). As the predator's reliance on the subsidization is increased (moving along increasing $w_{S}$ in Figure 2C, as long as S is sufficiently high), or as subsidy availability increases (moving along increasing S in Figure 2C), we observe the lower mass threshold of the predator to decline. This means that small‐predator body sizes above this declining threshold become feasible as the predator increasingly relies on the growing availability of subsidies. The same changes have an opposing impact on the herbivore's large‐size threshold due to the buoying effect subsidies have on the predator population, and the subsequent negative effects that predators have on herbivores (Figure 2D). As subsidy availability continues to increase, the large‐size threshold of the herbivore population declines until all size classes are infeasible (gray region in Figure 2D).

### Competition and prey‐switching

The inclusion of two competing herbivores without external subsidization in the competition model (Figure 1B), enriches the potential dynamics of the system, which vary with the relative body sizes of species as well as the flow of biomass between them. This motif comprises a predator with body size $M_{P}$, the predator's primary herbivore prey with population density $H_{1}$ and mass $M_{H_{1}}=E\left\{M_{H}|M_{P}\right\}$, a secondary herbivore prey with population density $H_{2}$ and mass $M_{H_{2}}=\phi M_{H_{1}}$, and the basal resource with density R. The primary herbivore prey always has a body size following the expected prey size for a given predator mass, while the secondary prey can vary with ϕ. When ϕ<1, the secondary prey is smaller than the primary prey; when ϕ>1, the secondary prey is larger. We note that $w_{1}$ is the proportion of predator growth from its primary prey, such that $w_{2}=\left(1-w_{1}\right)$ reflects its reliance on the secondary prey; $w_{1}\gg 0.5$ means that the predator has higher selectivity for its primary prey; $w_{1}\ll 0.5$ means that the predator has higher selectivity for its secondary prey. In contrast, $w_{1}\approx 0.5$ corresponds to low selectivity. Because of its status as primary prey, we may generally assume that $w_{1}>0.5$, though we show results for the full range.

**FIGURE 3:**
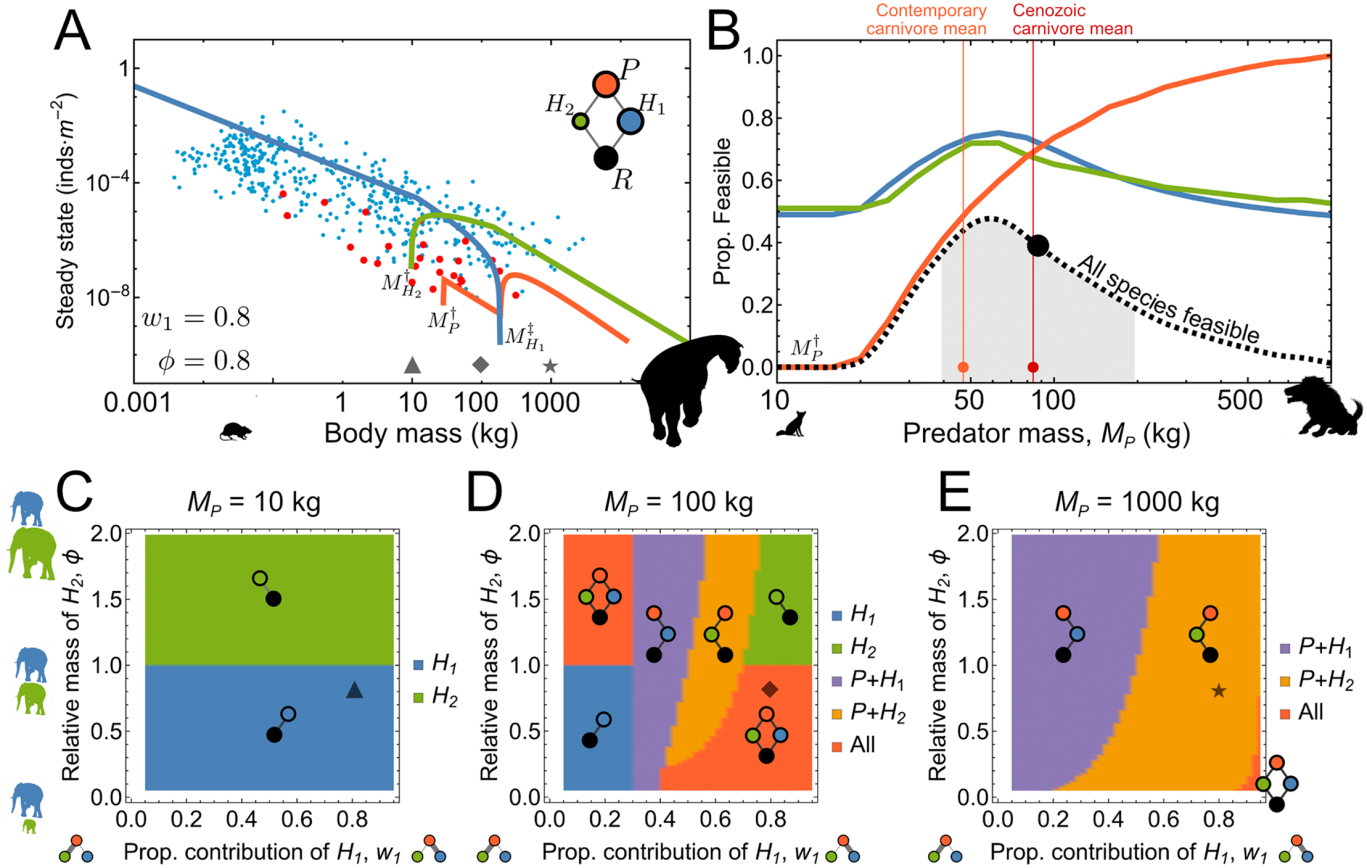
Population densities of species and the feasiblility of predators in the competition model. (A) Internal steady state densities for the predator and both herbivore prey, where the predator (red line) is specializing on the primary prey ($w_{1}=0.8$, blue line), with the secondary prey having a smaller body size (ϕ=0.8, green line). Empirical densities as in Figure 2. Symbols along the body mass axis relate to coordinates in the coexistence regions shown in bottom row: triangle = panel C ($M_{P}=10$ kg); diamond = panel D ($M_{P}=100$ kg); star = panel E ($M_{P}=1000$ kg). (B) Proportion of parameter space $\left(w_{1},\phi \right)$ resulting in feasible predator (red), primary prey (blue) and secondary prey (green) populations as a function of predator mass $M_{P}$. The predator mass where feasibility becomes zero is the predator lower mass threshold $M_{P}^{†}=20$ kg. The proportion of parameter space feasible for full coexistence is denoted by the dashed black line, which (normalized to a log‐mass probability distribution) gives a geometric mean of $M_{P}=87$ kg (black point), with the gray shaded region denoting ±1 standard deviation. Light and dark red points and vertical lines denote the empirical geometric mean body size of contemporary and Cenozoic carnivores at 47 and 84 kg, respectively, taken across species above the threshold size of 20 kg. (C–E) Coexistence of prey populations $H_{1}$, $H_{2}$, and the predator population P as a function of predator body mass (C) $M_{P}=10$ kg, (D) $M_{P}=100$ kg, and (E) $M_{P}=1000$ kg. The coexisting species are shown as full or partial motifs. Triangle/diamond/star symbols mark coordinates for body masses as in (A). Rodent silhouette obtained from www.publicdomainpictures.net (CC0 Public Domain); all others created by Justin D. Yeakel.

The competition model has 13 fixed points, but only one stable internal fixed point for a particular combination of predator and herbivore masses (Figure 3A). As with the subsidy model, the stability of the system is similarly bounded by a predator low‐mass threshold $M_{P}^{†}$ and a primary prey high‐mass threshold at $M_{H_{1}}^{‡}$. Importantly, this system additionally introduces a lower threshold for the secondary prey at $M_{H_{2}}^{†}$. In the example shown in Figure 3A, where $w_{1}=0.8$ and ϕ=0.8 (i.e., 80% of the predators' growth is from its primary prey, which is 25% larger than its secondary prey), predators with masses above the low‐mass threshold for the secondary prey ($M_{H_{2}}^{†}$) and below the high‐mass threshold for the primary prey ($M_{H_{1}}^{‡}$) have densities that are ratcheted to lower values. This is due to the combined effects of competition and preference for an increasingly scarce primary prey. Immediately beyond the high‐mass threshold for the primary prey, the predator population is buoyed by the secondary prey's release from competition.

The number of coexisting species depends on the body sizes of predators and herbivore prey. When the predator's mass is below the threshold $M_{P}^{†}\approx 20$ kg, the predator population is not feasible, leaving the two herbivore populations to compete directly (Figure 3B). In this case, competition leads to exclusion of the smaller herbivore species, such that the herbivore population $H_{1}$ excludes $H_{2}$ when ϕ<1, whereas $H_{2}$ excludes $H_{1}$ when ϕ>1 (Figure 3B,C). As the predator mass $M_{P}$ approaches 100 kg, the system straddles the lower mass and higher mass thresholds, where the coexistence of all species is possible (Figure 3B,D). In this region, predator selective preference for the larger prey (high $w_{1}$ and low ϕ, or low $w_{1}$ and high ϕ) limits the larger herbivore's fitness advantage, enabling coexistence of both herbivores with the predator (upper left and lower right corners of Figure 3D). The presence of all species in the motif defines a stable community—depicted by the black dashed curve in Figure 3B—and normalizing the curve to a log‐mass probability distribution gives a geometric mean of 87 kg (black point in Figure 3B, with the shaded region below the curve denoting ±1 standard deviation), with peak probability at 67 kg. If the predator prefers the smaller prey, both of these populations collapse, leaving the larger prey to persist alone (lower left and upper right corners of Figure 3D).

In contrast to these dietary extremes, predator non‐selectivity (w≈0.5) results in exclusion of the prey that contributes to a slightly larger proportion of predator growth, except when ϕ is low, in which case all species can coexist. For higher values of ϕ, this pattern is largely insensitive to the relative size of the secondary prey (along the ϕ axis; purple and yellow areas in Figure 3D,E). Here, the larger bodied predator has an expected prey with similarly larger body size, but where the lower reproductive rates of these larger prey cannot shield against the effects of a predator obtaining a significant portion of its growth from the secondary prey. This effect is similar to that of increased predator subsidization, lowering the herbivore upper threshold $M_{H}^{‡}$ and increasing the range of herbivore body sizes that are infeasible. It is thus the larger prey that is more likely to become the victim of increased predator subsidies, whether they arise from external resources or a secondary prey. This pattern expands across all $\left(w,\phi \right)$ as predators reach megapredator size classes ($M_{P}\to 1000$ kg; Figure 3E). The asymmetry in the boundaries between these steady states is likely due to (1) the super‐linear scaling of $E\left\{M_{H}|M_{P}\right\}$, and (2) the disproportionate spacing between ϕ>1 and ϕ<1, where the range of possible secondary prey masses is compressed below ϕ=1 and extended above it.

### Predator dietary selectivity

**FIGURE 4:**
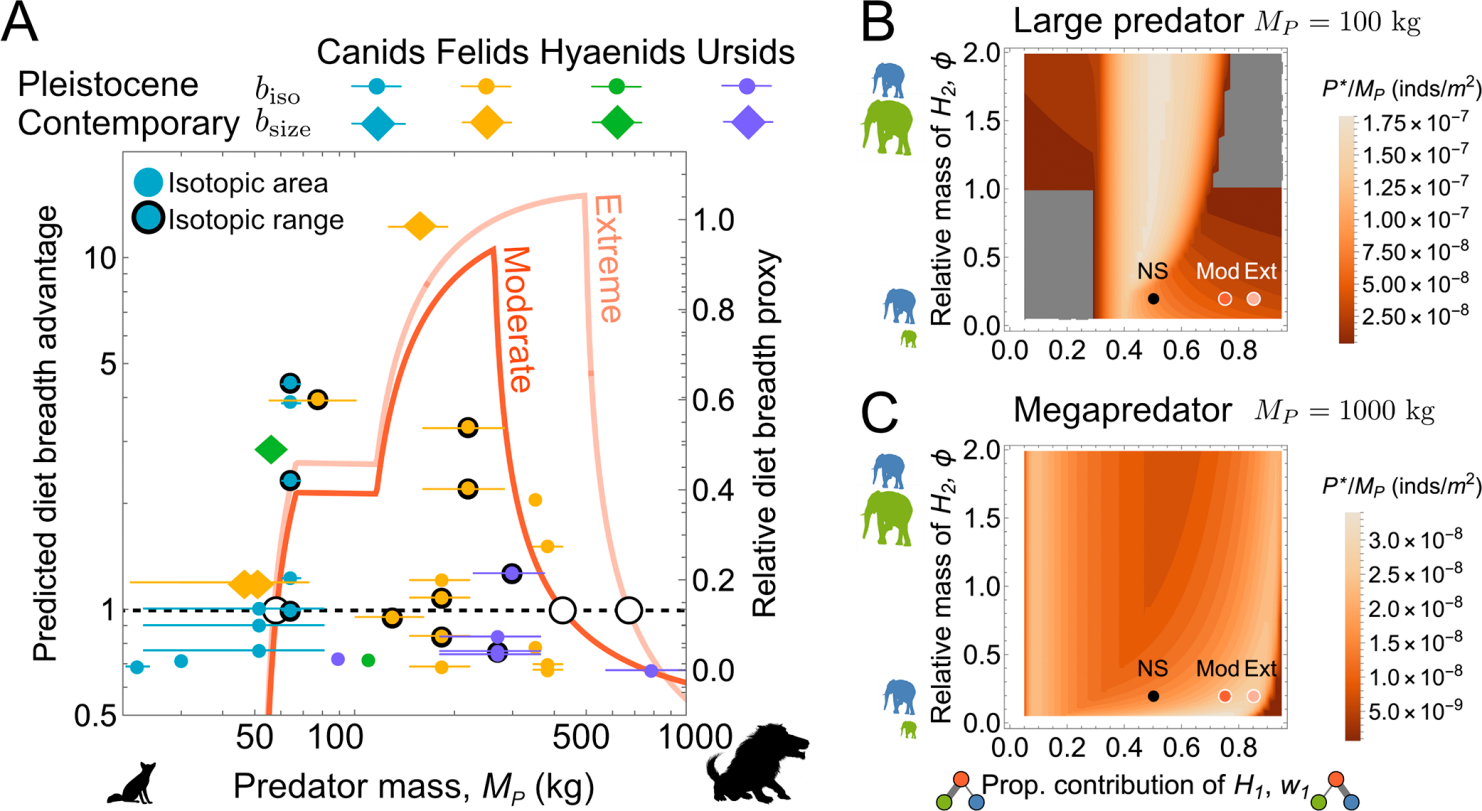
(A) The predicted dietary breadth advantage, measured as the ratio of non‐selective (NS; $w_{1}=0.5$) predator densities with higher dietary breadth to (1) moderately selective ($w_{1}=0.75$; dark red line) and (2) extremely selective ($w_{1}=0.85$; light red line) predator densities with lower dietary breadth ($P_{\mathrm{NS}}^{*}/P_{S}^{*}$; left *y*‐axis) as a function of predator mass $M_{P}$, where the relative size of the secondary prey is smaller than the primary prey (ϕ=0.2). A dietary breadth advantage exists for values of $P_{\mathrm{NS}}^{*}/P_{S}^{*}>1$. White circles along the dashed line denote where $P_{\mathrm{NS}}^{*}/P_{S}^{*}=1$. Colored points denote Pleistocene (small circles) and contemporary (large diamonds) relative diet breadth proxies ($b_{\mathrm{iso}}$ and $b_{\text{size}}$) as a function of predator size for canids (teal), felids (orange), hyaenids (green), and ursids (purple). Black outlined colored circles denote $b_{\mathrm{iso}}$ calculated from carbon isotopic range, while non‐outlined colored circles denote values calculated from carbon and nitrogen isotopic convex hull area. (B) and (C) Predator steady state densities $P^{*}/M_{P}$ (inds/m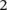) across $\left(w_{1},\phi \right)$ for (B) large ($M_{P}=100$ kg) and (C) megapredators ($M_{P}=1000$ kg). Black points represent the densities for the non‐selective (NS) diet, with red/orange points for moderate (Mod) and extreme (Ext) selective diets used in (A). Gray regions denote predator infeasibility for all size classes. Silhouettes created by Justin D. Yeakel.

We define predator dietary selectivity—in the context of the competition model—as a range from non‐selective, where diet breadth across the two alternative prey is maximized ($w_{i}\approx 0.5$), to an extreme selective preference for prey i ($w_{i}=1$). We emphasize that this definition highlights the relative importance of fueling growth from multiple mammalian prey of potentially different body sizes. The relative advantage of non‐selective (high breadth) versus selective (low breadth) diets, as a function of predator body size, can be evaluated by comparing the steady state densities of both. We quantify the predator's breadth advantage as the ratio of steady state densities between a non‐selective and selective predator ($P_{\mathrm{NS}}^{*}/P_{S}^{*}$) as a function of its body mass. We examine two scenarios: the breadth advantage of a non‐selective predator relative to a moderately selective predator ($w_{1}=0.75$), and that relative to an extremely selective predator ($w_{1}=0.85$), where we assume that the secondary prey is smaller than the primary prey (ϕ=0.2) such that the dietary breadth defined by non‐selectivity accords with a large range of prey body sizes. A value of $P_{\mathrm{NS}}^{*}/P_{S}^{*}>1$ thus means that the non‐selective dietary strategy benefits the predator population (high breadth advantage), while $P_{\mathrm{NS}}^{*}/P_{S}^{*}<1$ means the selective strategy is more beneficial (low breadth advantage). We observe that, in parameter regions where predators are feasible, there is a breadth advantage, relative to moderate selectivity, for predator size classes $57<M_{P}<420$ kg (dark red line, Figure 4A). This advantage extends to larger size classes when the breadth advantage is calculated with respect to extreme selectivity, where we observe a decline at ca. 668 kg (light red line, Figure 4A).

Our results show that the breadth advantage, with respect to either form of selectivity, declines as predator body size increases, giving way to a megapredator low breadth advantage (Figure 4A,C). In this low breadth advantage range, we observe that the megapredator drives its primary prey to extinction, subsisting instead on the smaller and more abundant secondary prey (Figure 3E). This effect is roughly symmetrical about $w_{1}$ and qualitatively invariant along ϕ, meaning that moderate selectivity on either the primary or secondary prey, regardless of the size difference between them, promotes the predator's steady state population density (Figure 4C).

To determine whether our predictions of the breadth advantage accord with estimates of diets from both contemporary and extinct large‐bodied (>20 kg) mammalian predators, we examine two separate proxies of relative dietary breadth. We specifically focus on predators specializing on vertebrate prey and do not include contemporary omnivores with observed diets diverging from vertebrate specialization. For extant predators, which include Serengeti cheetah (*Acinonyx jubatus*), leopard (*Panthera pardus*), hyaena (*Crocuta crocuta*), and lion, dietary breadth is measured directly as the ratio of the prey mass range consumed by each predator relative to the prey mass range available to all predators (Sinclair et al., 2003). In this case, relative dietary breadth is measured as the relative mass range of consumed prey, given by $b_{\text{size}}=q_{\text{pred}}/q_{\text{herb}}$, where $q_{\text{pred}}$ is the prey mass range utilized by individual predator species, and $q_{\text{herb}}$ is the prey mass range available to all predator species. For predators consuming a larger range of prey body sizes, $b_{\text{size}}\to 1$, implying dietary generalization. We observe this proxy of diet breadth to increase in good alignment with the predicted generalist advantage (Figure 4A).

Because contemporary mammalian predators are not representative of the full size range realized by predators in the fossil record for millions of years until very recently, we include 33 additional predator species from 14 separate assemblages spanning the early Pleistocene to the early Holocene, including—among others—the dire wolf (*Aenocyon dirus*), the American lion (*Panthera atrox*), saber‐toothed cats (*Smilodon* spp.), and the short‐faced bear (*Arctodus simus*), which is the largest included mammalian predator at ca. 780 kg (Coltrain et al., 2004; DeSantis et al., 2021; Feranec & DeSantis, 2014; Fox‐Dobbs et al., 2008; Fuller et al., 2014, 2020; Koch et al., 2004; Palmqvist et al., 2008; Trayler et al., 2015). While short‐faced bears are thought to have been megaherbivore specialists (Chatters et al., 2024; Fox‐Dobbs et al., 2008), included in our Pleistocene dataset are some smaller bodied ursids that may have been more omnivorous. For extinct species, dietary breadth is estimated from carbon isotope values (measured as $\delta^{13}C$) extracted from bone collagen or tooth enamel apatite for both predators and their potential herbivore prey, specific to each assemblage (see Appendix S1: Section S5). Relative diet breadth is then estimated as $b_{\mathrm{iso}}=r_{\text{pred}}/r_{\text{herb}}$, where $r_{\text{pred}}$ is the maximal isotopic range of each predator species, and $r_{\text{herb}}$ is the maximal isotopic range of all available herbivore species in a given assemblage. Where both $\delta^{13}C$ and $\delta^{15}N$ are available, $b_{\mathrm{iso}}$ is calculated from relative isotopic areas (measured as minimal convex hull area). If predators rely on the full suite of available prey (to different extents, among individuals and over time), their isotopic range is more likely to approximate that of the available herbivore community, such that $b_{\mathrm{iso}}\to 1$. Importantly, both $b_{\text{size}}$ and $b_{\mathrm{iso}}$ are strongly positively correlated when measured for contemporary mammalian predators. Moreover, while lower sample sizes will lead to underestimates of $b_{\mathrm{iso}}$, there is not a strong correlation between sample size and $b_{\mathrm{iso}}$ values measured either by isotopic range or area, such that sampling bias is not expected to impact the qualitative nature of our results (see Appendix S1: Section S5).

We observe that the isotopic proxy of diet breadth reveals much greater variability among extinct carnivores, though this variability is roughly contained within the predicted zone of the breadth advantage. At smaller size classes, we observe carnivores to have lower isotopic breadth, increasing for dire wolves and the similarly sized *Smilodon gracilis* along the predicted trajectory of the generalist advantage. At larger size classes, felids including other *Smilodon* spp., the American lion, and *Homotherium* spp. fill out the range of selectivity (low isotopic breadth) to non‐selectivity (high isotopic breadth). In contrast, ursids occupy a relatively smaller proportion of isotopic space implying comparative selectivity, including the only megapredator representative, the short‐faced bear.

## DISCUSSION

The effects of bioenergetic trade‐offs on the density and, by extension, feasibility of a species' population depend on its body size alongside the body sizes of those species in its local network of interactions (Brose, 2010; Rallings et al., 2024). Although such trade‐offs are known to structure the larger community through universal predator–prey biomass scaling laws (Hatton et al., 2015), the mechanisms linking individual bioenergetics to community‐level patterns remain elusive. We argue that bioenergetic bounds on population feasibility play a central role in shaping the fitness landscape contributing to larger scale trends in body size evolution.

### Subsidies, limits to predator specialization, and megaherbivore collapse

Alternative dietary subsidies apart from a species' primary prey arise from a variety of sources, including carcasses (Ritwika et al., 2024; Wilson & Wolkovich, 2011), plant material and invertebrates that complement diet and are abundant in the environment, resources from adjacent biomes (e.g., marine subsidies; Darimont et al., 2009, Pires & Galetti, 2023), and more recently anthropogenic activities (Hopkins III et al., 2014). Here, we assume that the subsidy simply represents a resource pool with density S that is dynamically unaffected to changes in the predator or prey populations over time. Our model predicts that a predator population specializing on a mammalian herbivore as prey is subject to a mass‐specific instability at body sizes between $M_{P}^{†}\approx 16$ and 22 kg (Figure 2). Importantly, this finding aligns with the observation that vertebrate‐feeding predators smaller than roughly 20 kg must subsidize their diet with additional resources (Carbone et al., 1999). We find that increasing subsidy availability, S, is required to enable ever smaller predator body sizes (lowering $M_{P}^{†}$; Figure 2C). Carbone et al. (1999) predicted a threshold for vertebrate‐specialist carnivores at ca. 20 kg based on the individual energetic limitations associated with hunting vertebrate prey as a function of predator body mass. Below this limit, predators have diets with significant contributions of non‐vertebrate resources, while those above tend towards vertebrate specialization. Our aligned prediction emerges from a population instability driven by bioenergetic trade‐offs, rather than individual foraging energetics, suggesting this approach captures fundamental features of natural systems.

The subsidization of predators has large implications for the survival of their herbivore prey. As has been previously demonstrated, increasing subsidy density promotes predator growth, ultimately driving herbivore populations to collapse (Holt, 2008; Nevai & Van Gorder, 2012). In this regime, there is not a cascading effect on the predator population because they are buoyed by the external subsidy. As both subsidy availability and the reliance of the predator on subsidies increase, an upper threshold emerges for the herbivore prey (Figure 2B,D), serving as an upper limit to body size. As subsidization increases, the upper threshold declines, limiting the range of feasible body sizes. This serves to destabilize the largest megaherbivores at the lowest subsidy densities, ultimately eliminating herbivore feasibility if subsidies to the predator increase enough (gray region, Figure 2D).

The implicit connection between the size‐specific stability of herbivore populations to predator subsidization may have direct implications for conservation planning (Pires & Galetti, 2023) as well as for understanding extinction events such as the end‐Pleistocene, which reveals demonstrable size selectivity in response to novel and subsidized predator pressure (Koch & Barnosky, 2006; Smith et al., 2018). Our results imply that even slightly increased predator reliance on prevalent subsidies can increase the extinction risk of the largest herbivores. In the context of the Pleistocene extinctions, this suggests that minimal reliance of human populations on alternative dietary resources (such as vegetation) could set the necessary conditions for the extinction of megaherbivores. That minimal subsidization dramatically lowers maximum feasible megaherbivore size classes, perhaps combined with sequential cascading effects across co‐occurring predators and their prey (Ripple & Van Valkenburgh, 2010), suggests that the bar for perturbing megafaunal communities may be lowered in the presence of subsidized predators such as humans.

### Fasting endurance and predator size evolution

Increasing the diversity and complexity of ecological communities can alter the outcome of interactions (Chesson & Kuang, 2008; Spaak & Schreiber, 2023). We observe that our competition model, which includes a predator and two competing herbivore prey (n=2, S=0), substantially increases the dynamical richness of the system. The emergence of three distinct body size thresholds (Figure 3A)—an upper threshold for the primary prey ($M_{H_{1}}^{‡}$), and lower thresholds for the predator ($M_{P}^{†}$) and secondary prey ($M_{H_{2}}^{†}$)—indicate that alternative community structures depend on specific combinations of species' masses and biomass flow (Figure 3C–E). These thresholds, which vary with dependence of the predator on each prey ($w_{1}$) and the relative size difference between prey (ϕ), combine to determine which species in the interaction motif can maintain positive population densities. While the positions of these mass thresholds change with the size difference of herbivores and the relative reliance of the predator on the primary prey, the presence of these thresholds is generally robust (Figure 3C,D), and appear even when $w_{1}$ is treated as a dynamic variable (Appendix S1: Section S4).

As with the subsidy model, we find that smaller bodied mammalian‐specialist predators (ca. $M_{P}<20$ kg) cannot maintain viable population sizes, even with multiple prey. As the predator is not externally subsidized in this case, this appears to be a hard limit reproduced by both frameworks (Figure 3B), again supporting Carbone et al. (1999). Smaller predators that are not viable become extinct, leaving the herbivores—which vary only in body size and the associated scaling of their vital rates—to compete with one another. As a result, the larger herbivore excludes the other given its increased starvation tolerance and lower sensitivity to declines in resource abundance (Figure 3C) (Rallings et al., 2024; Yeakel et al., 2018), mirroring observations of positive size selectivity resulting from resource competition (Bonner, 1988; Brown & Maurer, 1986; Kingsolver & Pfennig, 2004).

Considering more complex species interactions, as in the addition of a competing herbivore population, allows us to pinpoint additional body size thresholds within ecological communities. We observe that at $M_{P}\approx 100$ kg, coexistence of all species is possible as long as the predator preferentially targets the largest herbivore (Figure 3D), which serves to suppress the latter's inherent competitive advantage, thus enabling the smaller herbivore to maintain a foothold. Across parameter values for $\left(w_{1},\phi \right)$, the probability that all species in the tri‐trophic competition motif coexist increases and then decreases with predator mass, with a geometric mean at $M_{P}=87$ kg and a peak probability at $M_{P}=63$ kg (roughly the size of a spotted hyaena; Figure 3B). Predator and herbivore body sizes occupying this region tend to fall between the lower and upper body mass thresholds.

Our prediction of a predator mass giving rise to a maximally stable trophic structure—while specific to the competition model—aligns with the contemporary large‐bodied terrestrial carnivore body size average, with a geometric mean of ca. 47 kg (orange point in Figure 3B) (Smith et al., 2004), accounting for only terrestrial carnivores above the 20‐kg vertebrate‐specialization limit (Carbone et al., 1999). Notably, the contemporary geometric mean is less than that for terrestrial Carnivora across the Cenozoic at 84 kg (red point in Figure 3B) (Smith et al., 2003). This estimate sits very close to the predicted geometric mean predator mass associated with coexistence of all species in the competition motif at 87 kg. We suggest that this alignment between model prediction and observational data may point to a potential ‘attractor’ for mammalian predator body sizes, where the feasibility of a local neighborhood of interacting species is optimal. This body size range could be viewed as a stable core fueling the hypercarnivore ratchet described by Van Valkenburgh et al. (2004), where short‐term success attained by evolution beyond this core size range is followed by long‐term failure, and where the antecedents emerge again from this core to advance the next turn of the ratchet.

Our results show that stability of the interaction motif is maintained only if the predator specializes on the larger prey (red regions in Figure 3D), setting the stage for the selection of predator traits increasing the likelihood of successful acquisition and handling. Such positive size selection of the predator may, in turn, contribute to positive size selection within targeted herbivore clades to escape predation (Benton, 2002; Sinclair et al., 2003). At mega size classes, such an arms race would eventually hit the large‐mass herbivore threshold $M_{H}^{‡}$, perhaps an evolutionary expression of self‐organized criticality (Solé & Manrubia, 1996), where the herbivore prey is squeezed between the dual pressures of lower reproductive output and mortality induced by a predator large enough to specialize on it. These dynamics are expected to deteriorate in communities depleted of megafauna, for example through bottom‐up environmental change in the Pliocene (Faith et al., 2024) or top‐down overhunting in the Pleistocene (Koch & Barnosky, 2006), effectively decoupling coevolutionary feedbacks. In such contexts, the relative payoff of behaviors such as sociality and group hunting may become elevated and covary with size‐based energetics in nonlinear ways, though this merits a dedicated future investigation.

### Adaptive benefits of predator dietary breadth

Mammalian carnivores tend to incorporate a larger diversity of prey as they increase in body size (Gittleman, 1985), fueled in part by their access to larger spatial areas (Garland, 1983) and enhanced abilities to subjugate a larger range of prey size classes (Carbone et al., 2007; Sinclair et al., 2003). We evaluate the potential advantages of dietary selectivity directly, by comparing the relative densities of predators under the assumption of $w_{1}=0.5$ (non‐selective, high breadth diets), $w_{1}=0.75$ (moderately selective, lower breadth diets), and $w_{1}=0.85$ (extremely selective, low breadth diets). Where our model yields a crossover in the density advantage from non‐selective versus selective diets with increasing predator size, we interpret this as a population‐level mechanism biasing macroevolutionary trajectories first towards broader diets at intermediate/large‐size classes, and then narrower diets at extreme size classes. We emphasize that this crossover prediction emerges from the size‐dependent trophic dynamics and is thus well suited for comparison against empirical data. Throughout we hold the secondary prey to be smaller than the primary prey (ϕ=0.2) to ensure that non‐selectivity is synonymous with prey size breadth, and so that the full tri‐trophic motif persists at steady state (see Figure 3D), regardless of dietary selectivity.

Relative to moderately selective predators, we observe increasing advantages of non‐selective feeding (high dietary breadth) between predator body sizes $M_{P}=57$ kg and $M_{P}=421$ kg (Figure 4). Above this size range, we find that a more selective diet is advantageous. If the breadth advantage associated with a non‐selective diet is compared to extreme selectivity, the relationship reaches this change‐point at an increased $M_{P}=668$ kg. This suggests that the predicted advantages of dietary breadth begin to diminish at very large body sizes. Perhaps compellingly, if we remove the assumption of a constant $w_{1}$, instead allowing it to vary dynamically with the relative abundances of prey, we observe that selective megapredators also cause smaller transient oscillations in their prey populations (see Appendix S1: Section S4), providing additional support for this finding.

The expected breadth advantage closely aligns with empirical observations of prey size ranges for larger contemporary carnivores (Sinclair et al., 2003), where our metric of relative dietary breadth ($b_{\text{size}}$) increases sharply with Serengeti hyaena and lion (diamonds, Figure 4A). The incorporation of an isotopic proxy for dietary breadth ($b_{\mathrm{iso}}$) allows us to evaluate the potential alignment of Pleistocene carnivores, capturing a fuller picture of terrestrial megafaunal trophic interactions. Because $b_{\mathrm{iso}}$ is a rough proxy of diet breadth, it is prone to specialization bias. For instance, a smaller isotopic range or area may include many species of herbivore (e.g. multiple grazers consuming $C_{4}$‐photosynthetic grasses), such that a predator generalizing on many grazing herbivores may have a limited isotopic distribution that incorrectly prescribes a narrower dietary niche. The reverse bias is less likely to occur, given that a predator's isotopic spread on the order of that of the herbivore community is more likely to be a stronger signal of heightened dietary breadth. The distribution of $b_{\mathrm{iso}}$ values clearly demonstrates an increased potential for high dietary breadth among larger bodied carnivores, including saber‐toothed cats (*Smilodon* spp.) and the American lion (*Panthera atrox*), although these and other large‐bodied species across multiple assemblages run the gamut of dietary proclivities, including extreme selectivity. Of particular note is the largest carnivore, the short‐faced bear (*Arctodus simus*), and while it is only a single datum, it aligns with the predicted decline of the selective diet advantage at megapredator size classes.

Contemporary mammalian communities may engender a biased perspective on the form and function of past ecosystems (Søndergaard et al., 2025). Alongside the heightened diversity and larger sizes of megaherbivores in past communities (Faith et al., 2018, 2019), larger predators also reached mega‐size classes. Megapredators, including the mid‐Eocene *Andrewsarchus* (an artiodactyl), the late Eocene *Sarkastodon* (an oxyaenidont), and the mid‐late Miocene *Amphicyon ingens* (a bear‐dog), have estimated sizes ranging from 550 to 1000 kg (Burness et al., 2001; Sorkin, 2008). These species are perhaps outliers to the mammalian predatory condition, though species of more recent genera such as *Arctodus* and *Smilodon* have estimated sizes nearing 500–850 kg (Anyonge, 1993; Christiansen, 1999; Christiansen & Harris, 2005; Sorkin, 2006), so perhaps this condition is not so rare as might be assumed.

While little direct evidence exists for mammalian megapredator diets, the largest felids likely had variable dependence on both grazing and browsing herbivores (DeSantis et al., 2019; Feranec, 2004; Yeakel et al., 2013). Perhaps tellingly, isotopic evidence suggests that short‐faced bears in Beringia were largely specializing on caribou (*Rangifer tarandus*; Fox‐Dobbs et al., 2008, Yeakel et al., 2013), while those mid‐continent may have been specializing on mammoth (*Mammuthus columbi*; Chatters et al., 2024). Our theoretical framework predicts that species including saber‐toothed cats and the American lion would be capable of reaching similarly broad diets, if not more than those of contemporary lions, with perhaps only the largest megapredators such as *Arctodus*, *Sarkastodon*, and *Andrewsarchus* reverting to greater selectivity. This accords with biomechanical arguments, where it is thought that the extreme size of these megapredators would limit their potential prey to only the slowest and largest megaherbivores (Sorkin, 2008).

If megapredators tend towards dietary selectivity, one might expect a negative feedback where the most tenuous megaherbivore prey are driven to extinction by their specialist predators, perhaps triggering subsequent selection favoring smaller predators with broader diets. Such a process could act as an ecological ceiling on predator size (cf. Carbone et al., 2007) by way of transient, local prey collapses where large selective feeders are disadvantaged over the long term due to the vulnerability of megaherbivore prey (Rallings et al., 2024). The size‐dependent duration of hypercarnivores in the fossil record appears to support such a mechanism (Appendix S1: Section S6). While hypercanivore genera below 100 kg frequently achieve temporal spans ≥20 million years, those above 100 kg reach a maximum duration of ca. 4.7 million years. Of course, many ecological, evolutionary, and taphonomic mechanisms could contribute to this pattern, but its alignment with our dynamic predictions is notable. As a bioenergetic ceiling to predator size driven by limitations to prey stability would act in concert with bottom‐up pressures on size selection, how these forces combine could be complex. For most periods prior to the late Pleistocene, mammalian extinctions have been assumed to be driven by bottom‐up changes to the environment (Blois & Hadly, 2009; Faith et al., 2018, 2024). Given that top‐down drivers clearly impact contemporary ecosystems (Wallach et al., 2015), their role in shaping paleontological communities is of growing interest (Nascimento & Pires, 2025a; Ripple & Van Valkenburgh, 2010).

Recent work on the macroevolutionary consequences of predator–prey interactions suggests that extinction rates of predators can increase when prey availability declines (Nascimento et al., 2024; Nascimento & Pires, 2025b). For example, the loss of intermediate‐sized herbivores with the emergence of open habitats increased extinction risk for predators targeting those size classes (Nascimento et al., 2024). To what extent top‐down ecological forcing may introduce intrinsic limits on size and diet is less well understood, and whether such a process could leave measurable signatures in the fossil record is unclear. Along these lines, recent efforts have shown that felid evolution may have increased extinction rates in Antilocaprinae (which includes the contemporary pronghorn, *Antilocapra americana*), supporting the notion that top‐down pressures can directly shape macroevolutionary patterns (Nascimento & Pires, 2025a). While within‐community top‐down destabilization would be expected to yield a lagged sequence where the most vulnerable megaherbivores decline first, compared to bottom‐up destabilization producing more synchronous, guild‐wide herbivore attrition, evidence that could differentiate between the two is unlikely to be captured in most cases given the resolution of fossil preservation. While distinguishing between the two may be difficult, bioenergetic models may serve to provide insight into how such contrasting scenarios could translate into longer term ecological or evolutionary signals, which might be more likely to leave traces in the fossil record.

The dynamical richness of our framework increased dramatically with the addition of a second herbivore (Figure 3). It stands to reason that inclusion of bioenergetic rate laws into larger food webs (Valdovinos et al., 2023) may provide insight into larger patterns captured by long‐term trends in diversity (Pires et al., 2017, 2018; Yeakel et al., 2014). If communities are indeed shaped by top‐down pressures, we should expect repeated patterns of pulsed coextinctions, where (1) the evolution of megaherbivores favors the emergence of large specialized carnivores, (2) specialized predation suppresses megaherbivore populations, and (3) in turn elevating the extinction risk of megaherbivores and by extension their super‐sized specialist predators. More generally, to what extent species at different trophic levels and size classes might initiate or communicate size‐specific impacts across more distantly connected species may provide insight into larger scale consequences of herbivore or predator radiation or extinction events. We expect that transitioning from motif‐level mechanism to larger suites of interactions will enable a better understanding—and testable predictions—of how size and trophic position contribute to the shaping of macroevolutionary patterns across the Cenozoic.

## AUTHOR CONTRIBUTIONS

Justin D. Yeakel, Matthew C. Hutchinson, Christopher P. Kempes, and Mathias M. Pires conceived of and contributed to the development of the model. Paul L. Koch contributed original data. Justin D. Yeakel, Matthew C. Hutchinson, Christopher P. Kempes, Paul L. Koch, and Mathias M. Pires analyzed the results. Justin D. Yeakel, Matthew C. Hutchinson, Christopher P. Kempes, Paul L. Koch, Pedro D. S. Ugarte, Jacquelyn L. Gill, and Mathias M. Pires interpreted the results and contributed to the writing of this manuscript.

## CONFLICT OF INTEREST STATEMENT

The authors declare no conflicts of interest.

## Supporting information


Appendix S1.

## Data Availability

Data and code (Yeakel et al., 2026) are available in Zenodo at: https://doi.org/10.5281/zenodo.18842091.
