# Supplementary material for: Bioenergetic trophic trade‐offs determine mass‐dependent extinction thresholds across the Cenozoic
Source: Ecology. 2026 May 4;107:e70390. doi: 10.1002/ecy.70390 (PMC13137081; doi:10.1002/ecy.70390)
Supplement: Supplementary file 1 — Appendix S1. [file ECY-107-e70390-s001.pdf]

Appendix S1

Bioenergetic trophic trade-offs determine mass-dependent extinction thresholds across the Cenozoic

Justin D. Yeakel, Matthew C. Hutchinson, Christopher P. Kempes, Paul L. Koch, Pedro D.S. Ugarte, Jacquelyn L. Gill, Mathias M. Pires

Ecology

Section S1. Model details and allometric timescales

We use these two tri-trophic motifs to investigate and identify different energetic boundaries that determine the likelihood of population collapse as potential drivers of size selection. With the subsidy model, we aim to understand how increasing or decreasing the dynamic feedback between predator and prey (via tuning the predator's reliance on the subsidy) imposes size-specific energetic limitations on the trophic interaction. With the competition model, we aim to understand how the coupled feedback between the predator and competing herbivores alters or adds to these size-specific constraints. See main text Fig. 1 for an illustration of the two motifs.

The general tri-trophic food chain model is detailed in Eq. S1, with the full suite of parameters defined in Table S1. The growth of the predator population P is fueled by the mortality it inflicts on its prey proportional to $w_i\lambda_P/Y_P$, where w_i is the proportional reliance of the predator on herbivore i, λ_P is the predator's effective growth rate, and Y_P is the predator yield coefficient, or the grams of predator produced per gram of prey consumed. In the subsidy model, the growth of the predator is also fueled by an external subsidy S, proportional to w_S. We assume the predator's effective growth rate follows a linear (Type I) functional response across the herbivore population density H_i, and is maximized at $\lambda_{H_i}^{\max}$. The rate of consumption fueling the herbivore prey population(s) is proportional to the resource density and is given by $\lambda_{H_i}(R)/Y_{H_i}$, where $\lambda_{H_i}(R)$ is the effective herbivore growth rate and Y_{H_i} is the herbivore yield coefficient, or the grams of herbivore produced per gram of resource consumed (Kempes *et al.* 2012). We assume the herbivore's growth rate $\lambda_{H_i}(R)$ follows a saturating (Type II) functional response across the resource density R, where the maximum growth is $\lambda_{H_i}^{\max}$ and the resource half-saturation density is $\hat{k} = k/2$ (Rallings *et al.* 2024), where k is the resource carrying capacity. The resource grows logistically with growth rate α towards its carrying capacity, both of which are assumed to be characteristic of tropical grasses (see Table S1; Yeakel *et al.* 2018). Note that while the results in the main text and supplement are presented and reported in terms of body masses in kg, the allometries and derivations below are all premised in body masses in grams.

Predator mortality is the product of natural mortality μ_P, which is composed of both

TABLE S1: List of parameters, their meanings, and values or units.

Parameter	Description	Value/Units
P	Predator density	g/m^2
S	Constant subsidy density	g/m^2
H_1	Primary herbivore prey density	g/m^2
H_2	Secondary herbivore prey density	g/m^2
R	Plant resource	g/m^2
w_S	Predator reliance on subsidy	0:1
w_1	Predator reliance on primary prey	0:1
λ_P	Predator effective growth rate	1/s
Y_P	Predator yield coefficient	–
μ_P	Predator natural mortality rate	1/s
M_P	Predator body mass	g
n	Number of herbivore prey	
$\lambda_{H_i}(R)$	Herbivore i growth rate	1/s
Y_{H_i}	Herbivore i yield coefficient	–
$\lambda_{H_i}^{\max}$	Herbivore i maximum growth rate	1/s
μ_{H_i}	Herbivore i natural mortality	1/s
$\sigma_{H_i}(R)$	Herbivore i starvation mortality rate	1/s
M_{H_1}	Primary herbivore prey body mass	$\mathbb{E}\{M_H \vert M_P\}$ g
M_{H_2}	Secondary herbivore prey body mass	ϕM_{H_1} g
ϕ	Size scaling of the secondary prey	0.1:2.0
α	Resource growth rate	9.45×10^{-9} s^{-1}
k	Resource carrying capacity	2.3×10^4 g m^{-2}

initial cohort mortality and the cumulative effects of senescence (Calder III 1983, Rallings *et al.* 2024). Herbivore mortality includes the effects of natural mortality, μ_{H_i}, as well as starvation mortality $\sigma_{H_i}(R) = \sigma_{H_i}^{\max}(1 - R/k)$ – which is inversely proportional to resource density (Rallings *et al.* 2024, Yeakel *et al.* 2018) – and predation. The general tri-trophic system can then written as

$$\frac{\mathrm{d}}{\mathrm{dt}}P = \left(\sum_{i=1}^{n} w_i \lambda_P H_i + w_S \lambda_P^{\max} S\right) P - \mu_P P,$$
$$\frac{\mathrm{d}}{\mathrm{dt}}H_i = \lambda_{H_i}(R) H_i - \Big(\mu_{H_i} + \sigma_{H_i}(R)\Big) H_i - w_i \frac{\lambda_P}{Y_P} P H_i,$$
$$\frac{\mathrm{d}}{\mathrm{dt}}R = \alpha \left(1 - \frac{R}{k}\right) R - \sum_{i=1}^{n} \frac{\lambda_{H_i}(R)}{Y_{H_i}} H_i, \tag{S1}$$

where $\lambda_{H_i}(R) = \lambda_{H_i}^{\max} \frac{R}{\hat{k}+R}$ and $\lambda_P = \lambda_P^{\max}/Y_{H_i}k$. The rate laws describing the growth and mortality of both mammalian predator and herbivore species vary as a function of predator body mass M_P and herbivore body mass M_H. (Throughout, we will use the term M_H to refer to herbivore prey body size generally, and M_{H_i} to refer to prey species that are

specific to the subsidy and competition models.) We approach the derivation of vital rates with respect to predator and herbivore mass by solving for multiple timescales associated with ontogenetic growth, maintenance, and expenditure, based on the bioenergetic trade-offs associated with somatic growth and maintenance during ontogenetic growth. See the next section for our derivation of vital rates using this approach, also detailed in Yeakel *et al.* (2018) and Rallings *et al.* (2024).

The rate laws describing the growth and mortality of both predator and consumer vary as a function of predator body mass M_P and herbivore body mass H, representing a mammalian carnivore and herbivore, respectively. We approach the derivation of vital rates with respect to predator and consumer mass by solving for multiple timescales associated with ontogenetic growth, maintenance, and expenditure. The growth of an individual predator or consumer i from birth mass $m_i = m_0$ to its reproductive size $m_i = 0.95M_i$ (where M_i is its observed adult mass) is given by the solution to the general balance condition $B_0 m_i^\eta = E_m \frac{\mathrm{d}}{\mathrm{dt}} m_i + B_m m_i$ (West *et al.* 2001), where $E_m = 5774\ \mathrm{J} \cdot \mathrm{g}^{-1}$ is the energy needed to synthesize a unit of biomass (Hou *et al.* 2008, Moses *et al.* 2008), $B_0 = 0.047\ \mathrm{W} \cdot \mathrm{g}^{-\eta}$ is the metabolic normalization constant (Pirt 1965), B_m is the metabolic rate to support an existing unit of biomass, and the metabolic exponent $\eta = 3/4$ (West *et al.* 1997). Birth size is assumed to follow the allometric relationship $m_0 = 0.097M^{0.92}$ (Blueweiss *et al.* 1978, Stryer 1995). From this balance condition, the time required for an organism starting from mass m_i' to reach mass m_i'' follows

$$\tau(m_i', m_i'') = \ln\left(\frac{1 - (m_i'/M_i)^{1-\eta}}{1 - (m_i''/M_i)^{1-\eta}}\right) \frac{M_i^{1-\eta}}{a(1-\eta)}, \tag{S2}$$

where $a = B_0/E_m$. From this general equation, we calculate the timescale of reproduction for a predator or herbivore consumer of mass M_i as $t_{\lambda_i} = \tau(m_0, 0.95M_i)$, such that the reproductive rate is $\lambda_i^{\max} = \ln(\nu)/t_{\lambda_i}$, where $\nu = 2$ is the set number of offspring per reproductive cycle (Savage *et al.* 2004, Yeakel *et al.* 2018).

Yield coefficients for the predator and herbivore are given by $Y_i = M_i \epsilon(M_i) E_d / B_{\lambda_i}$ and represent the grams of consumer produced per gram of resource consumed. Here, B_{λ_i} [J] is the lifetime energy use required to reach maturity ($B_{\lambda_i} = \int_0^{t_{\lambda_i}} B_0 m_i(t)^\eta \mathrm{dt}$), E_d [J/g] is the energy density of that which is being consumed (Yeakel *et al.* 2018), and the foraging efficiency ratio $\epsilon(M_i) = \epsilon_0 M_i^{\epsilon_1}$ is the proportion of that energy available to the consumer.

Gathering prefactors into $\kappa = \epsilon_0/B_0$, we write

$$Y_i = \kappa \frac{E_d M_i^\eta}{\int_0^{t_{\lambda_i}} m_i(t)^\eta \mathrm{dt}}. \tag{S3}$$

In this formulation, κ is a constant that converts dietary energy density into per-gram yield relative to the integrated metabolic cost to maturity. When κE_d is large, energetic resources are efficiently converted to biomass, which may occur when assimilation is unconstrained, gut processing is efficient, or for lower-cost metabolic machinery as occurs in ectotherms. When κE_d is small, energetic resources are less efficiently converted to biomass, as may be the case when consumers forage on poor-quality or well-defended resources. Throughout we assume that $\kappa \approx 1$, which interacts with resource growth rate and carrying capacity to calibrate mass-density intercepts near those observed for herbivores and carnivores (Carbone & Gittleman 2002, Damuth 1987).

While E_d changes with the type of resource being consumed (i.e. plant versus animal tissue – see below), throughout we assume that the foraging efficiency ratio $\epsilon(M_i) = \epsilon_0 M_i^{\epsilon_1}$, and $\epsilon_1 \approx -1/4$. For herbivores, $\epsilon(M_i)$ represents a population-level realized access term that combines resource availability and interception. An ecologically-motivated scaling of assimilation efficiency is given by imagining herbivore individuals sweeping through a resource area with energy density ρ_R [J/m^2], and with interception/capture of the resource defined by its velocity $v(M_H) \propto M_H^{0.13}$ [m/s] and reaction distance $d(M_H) \propto M_H^{1/3}$ [m] (together, the expected supply). Taken relative to the herbivore's metabolic demands $B_H \propto M_H^{3/4}$ [J/s], we get $\epsilon_H(M_H) = 2\rho_R v d/B_H \propto M_H^{-0.28} \approx M_H^{-1/4}$. Together, this results in an approximate $-1/4$ exponent for the scaling of yield.

A separate check on the $-1/4$ scaling of yield is obtained by comparing the consumer response in our framework to that of the Holling Type II functional response. While our parameterization is rooted in supply-coupled mechanics, where consumer response is a function of resource availability, Holling Type II kinetics require the enumeration of handling time and search rate, which are perhaps more ambiguous in the deep past, and particularly so among mega-size classes that are no longer represented in terrestrial environments. Nevertheless, we can contextualize our parameterization in these terms, and find that $Y_i = t_h M_i \lambda_i^{\max}$, where t_h is handling time per gram of resource and is empirically found to scale as $M^{-1.02}$ in 2D environments (Pawar *et al.* 2012). This equivalence means that, at saturation, production is limited by how fast a consumer can process food (where throughput is $1/(M_i t_h)$).

Given that maximal reproductive growth scales with a $-1/4$ exponent (Yeakel *et al.* 2018), it follows that $Y_i \propto M_i^{-1/4}$.

For predators, the $-1/4$ scaling of yield can be recovered by taking into account the limitations of consumer gut capacity given the increasingly large body size of prey with increasing predator size, and the notion that larger predators can better defend, and return to, their kills. As such, we define $q(M_P)$ as the decreasing proportion of prey available to predator consumption with increasing size, which can be expressed as $q(M_P) = \chi(M_P)N_e(M_P)$, where $\chi(M_P)$ is the per-bout fraction of prey mass that fills a predator's gut (G/M_H), and $N_e(M_P)$ is the number of bouts available to a predator as the kill is subject to external decay and kleptoparasitism. With gut capacity $G(M_P) \propto M_P^1$ and prey body size $M_H \propto M_P^{1.46}$, we observe $\chi(M_P) \propto M_P^{-0.46}$. The number of successive bouts declines over time, and if $\psi(M_P)$ is the probability that the carcass remains, then N_e can be written $N_e(M_P) = 1 + \psi(M_P) + \psi(M_P)^2 + ... = (1 - \psi(M_P))^{-1}$. We then assume that $\psi(M_P) = \exp\{-\lambda_{\text{loss}}(M_P)\tau(M_P)\}$, where $\lambda_{\text{loss}}(M_P) \propto M_P^{-\xi}$ is the per unit time rate at which a predator's kill becomes unavailable, and $\tau(M_P) \propto M_P^{0.25}$ is the predator's mean digestive retention time (the time required to clear the gut). The probability $\psi(M_P)$ is therefore a survival function, which we assume approaches unity more rapidly with increasing predator body size because it is assumed that larger predators can better defend their prey. So larger predators have a lower per-unit-time rate that a kill becomes unavailable, which (for $\xi > 0.25$) makes $\lambda_{\text{loss}}(M_P)\tau(M_P)$ smaller and therefore pushes $\psi(M_P)$ closer to unity. If $\psi(M_P)$ is the probability that a carcass remains following successive bouts, the number of bouts available to a predator ($N_e = (1 - \psi(M_P))^{-1}$) can be approximated as $N_e \approx (\lambda_{\text{loss}}(M_P)\tau(M_P))^{-1}$ if $\lambda_{\text{loss}}(M_P)\tau(M_P) \ll 1$. This approximation implies that the carcass loss is slow relative to the predator's feeding cycle, and that a predator typically gets multiple returns before the kill is gone.

Taken together, we observe the exponent of $q(M_P)$ to be approximately $q_1 = -0.46 + (\xi - 0.25)$ (using the small $\lambda_{\text{loss}}\tau$ approximation for N_e above). The decline of the proportion of individual kills available to predators can be combined with the expected supply relative to metabolic demand in the same way as was done for herbivores to get $\epsilon_P(M_P) = \rho_H \alpha(M_P) q(M_P)/B_P$, where ρ_H is the energy density of prey on the landscape (and is independent of prey mass, given energetic equivalence arguments), $\alpha(M_P) \propto M_P^{0.63}$ is the search rate of predators foraging for dynamic prey in a 2D environment, and $B_P \propto M_P^{3/4}$

is the metabolic rate of the predator. This results in $\epsilon_P(M_P) \propto M_P^{-0.12+q_1}$. If yield, and by extension $\epsilon_P(M_P)$, is assumed to have an exponent of $-1/4$ as is implied from the production at saturation equivalency shown above, we would then find that the (unknown) exponent associated with the rate of kill loss would need to be $\xi = 0.58$, such that $q_1 = -0.13$. If we set $\xi = 0.58$, this would mean that a 10-fold increase in predator size would result in a $\approx 2.13\times$ increase in the number of times a predator returns to its kill to fill its stomach. Compared to a 20 kg predator (e.g. a large coyote or lynx), which we assume would fill its stomach with one kill, a 200 kg lion would be expected to fill its stomach $2.13\times$ from a single kill. This lines up with expectations from observations of lions in East Africa, where kills are maintained for 10-16 hours for typical prey (zebra on the low end, buffalo on the high end) (Scheel 1993). Based on gut passage times of 6 to 8 hours, this translates to between 2 and 3 feeding cycles per individual, which is in line with our expectation for N_e.

Following Rallings *et al.* (2024), the energy density of herbivore consumers changes with body mass M_H. For example, small mammals have very low percent body fat, whereas very large mammals have high percent body fat. Because the amount of consumable tissues with different energy densities within an herbivore varies allometrically, so too should the energy density of herbivore prey $E_d(M_H)$. We consider four primary tissue groups: a consumable set composed of muscle, fat, and other tissues, and a non-consumable set composed only of skeletal tissues. If the scalings associated with fat, muscle, and skeletal tissues are $M_H^{\text{fat}} = f_0 M_H^{1.19}$, $M_H^{\text{musc}} = g_0 M_H^{1.00}$, and $M_H^{\text{skel}} = h_0 M_H^{1.09}$ (Prange *et al.* 1979), the scaling of the *other* tissue (gut tissue, organ tissue, etc) is given by $M_H^{\text{other}} = M_H - (M_H^{\text{fat}} + M_H^{\text{musc}} + M_H^{\text{skel}})$. The energy density of fat is $E_{\text{fat}} = 37700$ J/g, whereas the energy density of muscle is $E_{\text{musc}} = 17900$ J/g (Merrill & Watt 1973). If we assume that gut and organ tissues have roughly the same energy density as muscle, the attainable energy density for an herbivore of size M_H is given by

$$E_d(M_H) = E_{\text{fat}} \frac{M_H^{\text{fat}}}{M_H} + E_{\text{musc}} \left(\frac{M_H^{\text{musc}}}{M_H} + \frac{M_H^{\text{other}}}{M_H} \right). \tag{S4}$$

To determine the maximum rate of mortality due to starvation $\sigma_H^{\max}$, we calculate the time required for a consumer to metabolize its endogenous energetic stores, estimated from its cumulative fat and muscle mass (Rallings *et al.* 2024, Yeakel *et al.* 2018). During starvation, we assume that an organism burns its existing endogenous fat stores as its sole energy source, such that the balance condition is altered to $\frac{\mathrm{d}}{\mathrm{dt}} m_i E'_m = -B_m m_i$, where $E'_m = 7000\ \mathrm{J} \cdot \mathrm{g}^{-1}$

is the amount of energy stored in a unit of biomass (Dunbrack & Ramsay 1993, Pirt 1965), which differs from the amount of energy used to synthesize a unit of biomass E_m (Yeakel *et al.* 2018). The natural mortality rate μ_i is derived by assuming that population cohorts decline over time following a Gompertz relationship (Calder III 1983). Using mammalian allometric relationships for the average initial cohort mortality rate, the actuarial mortality rate, and organismal lifespan, we obtain μ_i as a function of body mass M_i (Rallings *et al.* 2024).

The natural mortality rate is obtained by first assuming that the number of surviving individuals in a cohort N follows a Gompertz relationship (Calder III 1983), where

$$N = N_0 \exp\left(\frac{q_0}{q_a}\Big(1 - \exp(-q_a t)\Big)\right), \tag{S5}$$

given that q_0 is the initial cohort mortality rate, and q_a is the annual rate of increase in mortality, or the actuarial mortality rate. The change in the cohort's population over time then follows

$$\frac{\mathrm{d}}{\mathrm{dt}} N = -dN, \tag{S6}$$

such that

$$d = -\frac{1}{N}\frac{\mathrm{d}}{\mathrm{dt}} N. \tag{S7}$$

If t_ℓ is the expected lifetime of the organism, then the average rate of mortality over a lifetime t_ℓ is

$$\begin{aligned}\mu &= \frac{1}{t_\ell}\int_0^{t_\ell} q_0 \exp(q_a t_\ell) \\ &= \frac{q_0}{q_a t_\ell}\Big(\exp(q_a t_\ell) - 1\Big).\end{aligned} \tag{S8}$$

The cohort mortality rate q_0, the actuarial mortality rate q_a and the expected lifetime t_ℓ of a mammal with mass M_i all follow allometric relationships, where $q_0 = 1.88 \times 10^{-8} M_i^{-0.56}$ (1/s) and $q_a = 1.45 \times 10^{-7} M_i^{-0.27}$ (1/s) assuming M_i is in grams. Together, we obtain the allometric relationship

$$\mu(M_i) = \frac{3.21 \times 10^{-8}\left(\exp(0.586 M_i^{0.03}) - 1\right)}{M_i^{0.59}}. \tag{S9}$$

Section S2. Predator-prey mass relationship

To obtain the expected prey mass for a given predator mass $\mathbb{E}\{M_H|M_P\}$, we combined mammalian trophic relationships documented in the FoRAGE database (Uiterwaal *et al.*

2018) with those for larger mammalian predators documented in numerous publications by Hayward et al. (Hayward 2006, Hayward *et al.* 2006a,b, Hayward & Kerley 2008, 2005, Hayward *et al.* 2006c). While previous accounts have estimated the mammalian PPMR to have a slope (in log-log space) closer to unity, the inclusion of trophic relationships for larger predators and prey, which are weighted by prey preference, suggests a slope closer to 1.46, which is the predator-prey mass relationship (PPMR) used in our analyses (Fig. S1). Exclusion of grizzly bears (*Ursus arctos*, which have a very low expected prey mass due to their specialization on salmon) elevates the slope to 1.5, though we note that such minor changes to the PPMR do not impact our findings. The expectation used throughout is set as $\mathbb{E}\{M_H|M_P\} = 3.73 \times 10^{-6} M_P^{1.46}$.

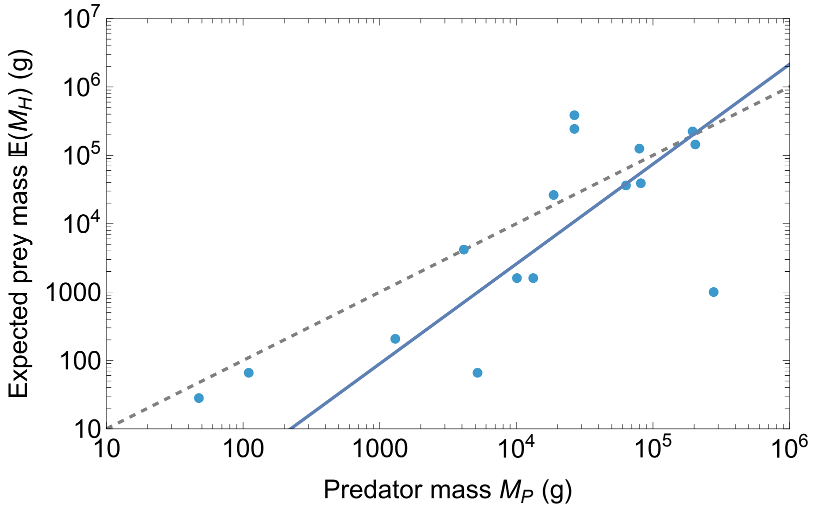

FIG. S1: The expected prey size for a given predator size $\mathbb{E}\{M_H|M_P\}$. The gray dashed line denotes the linear relationship described by Carbone et al. (Carbone *et al.* 1999), and the blue line denotes the fitted relationship (slope: 1.46) using the data sources described in the main text.

Section S3. Predator subsidization thresholds

The lower-mass predator threshold $M_P^\dagger$ defines the minimum predator body size that can sustain a positive population density. Under the conditions of subsidization, we examine the frequency distribution of this lower-mass threshold when the predator is specializing on mammalian prey (such that $w_S < 0.1$). The lower-mass threshold distribution (Fig. S2) thus defines a mammalian predator's lower body size limit when it specializes on mammalian prey.

This distribution has a peak mode at $M_P^\dagger = 22$ kg, and a long-tail towards lower $M_P^\dagger$ values, with a mean of 15 kg. Predators with a body size lower than this threshold mass require increased subsidization to support their populations. This cut-off aligns closely with the vertebrate-specialization threshold predicted and measured by Carbone et al. (Carbone *et al.* 1999) at ca. 21 kg. Importantly, the prediction of the vertebrate-specialization threshold was obtained using energetic constraints based on intake rates, such that our dynamic population approach offers independent verification of this trophic constraint for mammalian predators.

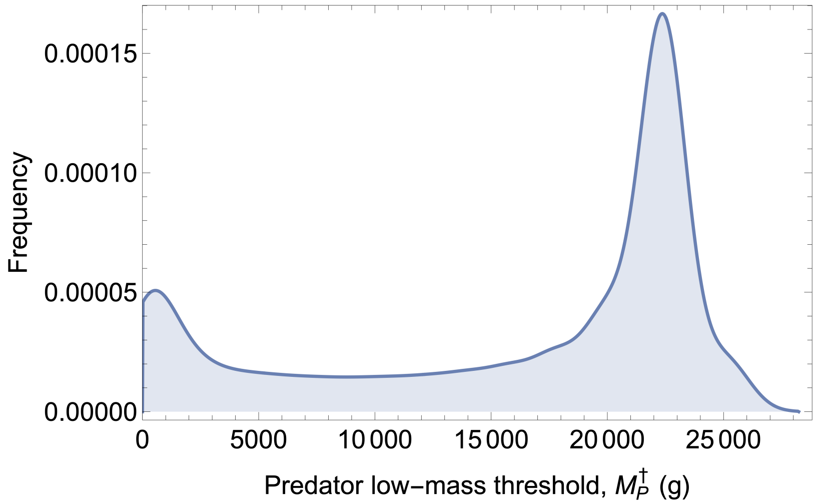

FIG. S2: Frequency distribution of the low mass predator threshold $M_P^\dagger$ when dependence on the subsidy is low, such that $w_S < 0.1$, meaning that the predator is primarily relying on the herbivore prey. The average low mass threshold under these conditions is $\langle M_P^\dagger \rangle = 15$ kg, with the primary mode at $M_P^\dagger = 22$ kg.

Section S4. Density-dependent switching

We have assumed throughout that the proportional contribution of the primary prey to the predator (w_1) is constant, such that short-term fluctuations in foraging are set aside to focus on long-term, average effects on species survival and reproduction. So while year-to-year changes in predator foraging respond to immediate changes in prey populations, it is the prey dynamics over decades and centuries shaping the average predator response, and this scale at which macroevolutionary insight is obtained. As such, we suggest it is the averaged response of a foraging strategy, typified by the exclusion of shorter-term ecological

fluctuations, that better captures the dynamics at work in shaping constraints on macroevolutionary process. However, we may ask to what extent our findings hold if we assume that the proportional contribution of each herbivore species changes in direct proportion to their abundances, which should not be confused with a true adaptive response (Kondoh 2003, Valdovinos *et al.* 2023, 2010). As such, we can allow $w_1 = \nu H_1 / \sum_j H_j$ where we interpret ν to be the responsiveness to relative changes in primary prey abundance. We observe that the inclusion of density-dependent prey-switching does not substantially change the dynamic regimes introduced by the competition model, with both predator low-mass thresholds $M_P^\dagger$ and consumer high-mass thresholds $M_H^\ddagger$ readily apparent (Fig. S3). Notably, density-dependent prey-switching does elevate predator population densities far above the observed mass-density relationship, as the predator is effectively feeding on a cumulatively larger prey reserve.

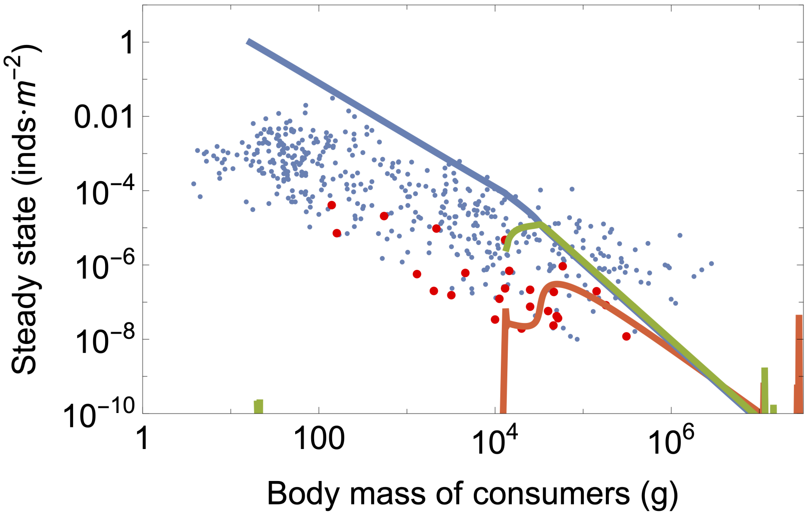

FIG. S3: The mass-density relationship for the primary and secondary herbivore (blue and green, respectively), and the predator (red). Densities are elevated but follow a similar scaling as the tri-trophic competition model (refer to Fig. 2a, main text).

The size-dependent benefits of increased predator dietary non-selectivity (increased dietary breadth) can also be observed when w_1 is itself a product of the relative abundance of prey. Because in this case, w_1 is no longer a fixed value, we are unable to directly distinguish selectivity from non-selectivity in the same way as in the main text. Instead, we might surmise that a non-selective (NS) predator is more responsive to changes in relative prey abundance, whereas a selective (S) predator is less responsive, such that $\nu_{\rm NS} > \nu_{\rm S}$. For ex-

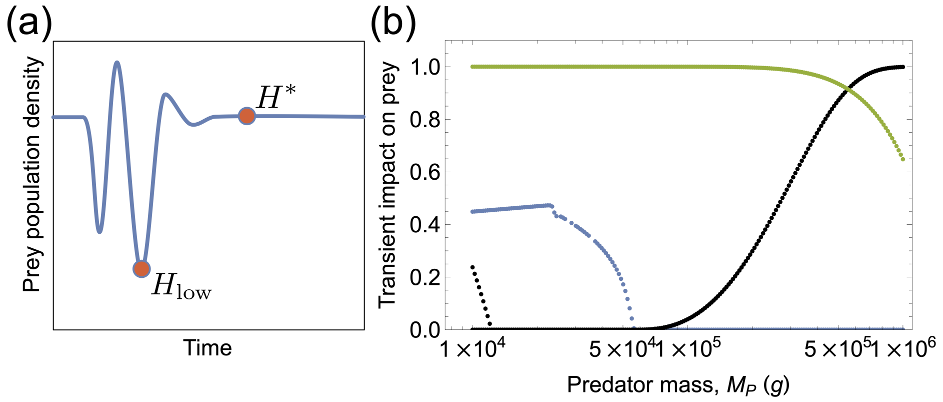

FIG. S4: (a) The transient oscillations of an herbivore prey with respect to a non-selective or selective predator, where the lowest density is denoted as $H_{\rm low}$, and the post-transient steady state density is denoted as H^*. (b) The impact of a non-selective predator versus selective predator on the transient dynamics of the primary and secondary herbivore prey. A non-selective predator is defined here by one that has a high rate of prey-switching, such that $\nu_{\rm NS} > \nu_{\rm S}$. The effect of the non-selective predator on both herbivore prey as a function of its body size is shown in black; the effect of a selective predator differs across prey, and is shown for the primary prey in blue and the secondary prey in green. Transient impact is defined in Equation S10. As the predator body mass increases, the non-selective predator (black line) tends to have a lesser impact on the transient dynamics of its prey until the change-point body size of $M_P = 549$ kg, above which the specialized predator has a lower transient impact.

ploratory purposes here, we will assume that the non-selective predator has a prey-switching rate 10× that of a selective predator.

Dynamic predator diets typically generate extreme transient fluctuations (Kondoh 2003), where the risk that a large fluctuation induces extinction can be significant. We measure the effect that different predators have on the transient dynamics of their prey as the transient impact

$$\mathcal{I} = 1 - \frac{H_{\rm low}}{H^*}, \tag{S10}$$

where H^* is the post-transient steady state of the herbivore population density, and $H_{\rm low}$ is the lowest transient condition of the fluctuating herbivore population prior to settling to the steady state. If the transient drop in herbivore population density is extreme, $H_{\rm low} \to 0$ and $\mathcal{I} \approx 1$, meaning that the impact is maximal and will likely result in the elimination of

the prey population. If the transient drop in the herbivore population is minimal, $\mathcal{I} \approx 0$.

We find that non-selective predators relative to selective predators produce smaller transients in their prey for predators up to the body size $M_P = 549$ kg, above which selective predators generate a lower transient impact (Fig. S4). Importantly, the change-point marking a switch from a non-selective to selective advantage is similar to that measured for extreme selectivity in the non-adaptive framework presented in the main text (530 kg). While the predator mass marking the upper limit of the non-selective (high dietary breadth) advantage in the main text and that based on relative transient impact are very similar, we suggest that this quantitative agreement is not inherently meaningful, as the change-point can be manipulated by changing the degree of selectivity (when w_1 is a set value), and the relative difference of the responsiveness assumed for non-selective and selective predators (when w_1 is dynamic). Instead, what is significant is the qualitative alignment that different non-selective, high dietary breadth advantages sunset at extreme predator body sizes, above which selectivity lowers the likelihood of a trophic-induced instability. These results suggest that there may be multiple dynamical effects at play, serving to differentiate the impact that megapredators have on their herbivore prey relative to their smaller-bodied evolutionary cousins.

Section S5. Empirical estimates of predator dietary breadth

We calculate empirical proxies for predator dietary breadth in two ways for contemporary and extinct predators. As described in the main text, for extant predators, which include Serengeti cheetah, leopard, hyaena, and lion, dietary breadth is measured directly as the ratio of the prey weight range consumed by each predator relative to the prey weight range available to all predators (Sinclair *et al.* 2003). In this case, relative dietary breadth is given $b_{\text{size}} = q_{\text{pred}}/q_{\text{herb}}$, where q_{pred} is the prey mass range available to individual predator species, and q_{herb} is the prey mass range available to all predator species. For predators consuming a larger range of prey body sizes, $b_{\text{size}} \to 1$, implying lower dietary selectivity.

To estimate dietary breadth for extinct species, we include 33 additional predator species from 14 separate assemblages spanning the early Pleistocene to the early Holocene (Coltrain *et al.* 2004, DeSantis *et al.* 2021, Feranec & DeSantis 2014, Fox-Dobbs *et al.* 2008, Fuller *et al.* 2014, 2020, Koch *et al.* 2004, Palmqvist *et al.* 2008, Trayler *et al.* 2015), including

the dire wolf (*Aenocyon dirus*), the American lion (*Panthera atrox*), saber-toothed cats (*Smilodon* spp.), and the short-faced bear (*Arctodus simus*), which is the largest included mammalian predator at ca. 780 kg. Body size estimates for all predators were gathered from the literature (Anyonge & Roman 2006, Christiansen & Harris 2005, Dantas 2022, Figueirido *et al.* 2011, Flower 2016, Gazin 1942, Hill & Easterla 2023, Koufos *et al.* 2018, Marciszak & Lipecki 2022, Palmqvist *et al.* 1996, 2002, Sherani 2016, Sorkin 2006) and are specified in detail in Supplementary Data (Yeakel *et al.* 2025).

For these extinct species, dietary breadth is estimated in one of two ways. We collected carbon and nitrogen isotope values (measured as δ^{13}C and δ^{15}N) extracted from bone collagen (protein) or carbon isotope values only from tooth enamel (bioapatite carbonate) for both predators and their potential herbivore prey, specific to each assemblage. Assemblages for which both carbon and nitrogen isotope values were obtained were from environments dominated by C_3 vegetation, whereas those for which only carbon isotope values were obtained were from mixed $C_3 - C_4$ environments. We estimated relative diet breadth for predators occupying C_3 environments from the relative size of isotopic convex hull area of the predator (CHA; in 2D carbon-nitrogen isotopic space), such that $b_{\rm iso} = {\rm CHA}_{\rm pred}/{\rm CHA}_{\rm herb}$, where ${\rm CHA}_{\rm pred}$ is the convex hull area of each predator species, and ${\rm CHA}_{\rm herb}$ is the convex hull area of all available herbivore species in a given assemblage. We note that convex hull area is used in lieu of standard ellipse area (calculated from isotopic covariance), or Bayesian ellipse area (taking into account the effects of sample sizes) (Jackson *et al.* 2011), to reduce the potential for over-estimating predator non-selectivity relative to the herbivore community. Where predator species were represented by < 3 individuals, convex hull area could not be calculated, and the maximal δ^{13}C range was used instead, such that $b_{\rm iso} = r_{\rm pred}/r_{\rm herb}$, where $r_{\rm pred}$ is the maximal δ^{13}C range of each predator species, and $r_{\rm herb}$ is the δ^{13}C isotopic range of all available herbivore species in a given assemblage. In mixed $C_3 - C_4$ environments, the relative diet breadth of predators is similarly estimated as $b_{\rm iso} = r_{\rm pred}/r_{\rm herb}$.

To ensure that both size-based ($b_{\rm size}$) and isotope-based ($b_{\rm iso}$) measures of dietary breadth capture similar tendencies, we evaluated both for contemporary carnivores occupying mixed $C_3 - C_4$ environments in East Africa. Using $b_{\rm size}$ measured in Sinclair *et al.* (2003) for Serengeti cheetah, leopards, wild dogs, hyenas, and lions, we compared these values against $b_{\rm iso}$ of the same species using an unpublished dataset of contemporary East African δ^{13}C values. We find a strong positive correlation between both measures, with wild dogs as the

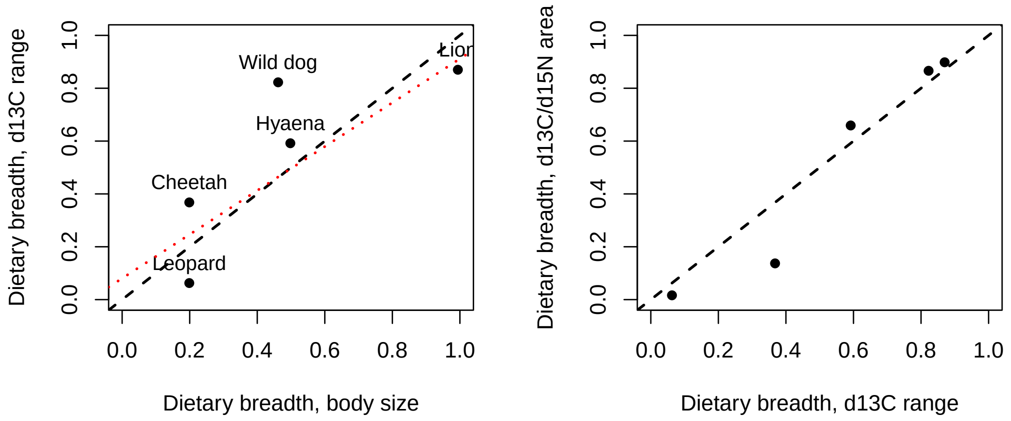

FIG. S5: (a) A comparison of dietary breadth estimated from relative body size ranges (b_{size}) versus that estimated from δ^{13}C values (b_{iso}) for contemporary carnivores in East Africa. The dashed black line denotes the 1:1 line. The dotted red line shows the best linear model fit when wild dogs are excluded ($R^2 = 0.75$, $p = 0.08$) (b) A comparison of dietary breadth measured from both δ^{13}C range and $\delta^{13}\text{C} + \delta^{15}\text{N}$ area ($R^2 = 0.92$, $p = 0.007$).

only clear outlier (Fig. S6a). With wild dogs included, $R^2 = 0.52$, and $p = 0.10$. With wild dogs excluded, $R^2 = 0.75$, and $p = 0.08$. Wild dogs are known to have an enlarged dietary range due to their efficient cooperative hunting behaviors (Creel & Creel 2002), so a magnified isotopic range is not entirely surprising. While neither relationship is statistically significant, the strong positive correlation – with or without wild dogs – supports the notion that these independent measures of breadth are, in principle, aligned. Dietary breadth inferred from stable isotopes is well-captured both in terms of δ^{13}C range and in $\delta^{13}\text{C} + \delta^{15}\text{N}$ area (Fig. S6b).

Finally, accurate measurement of dietary breadth from stable isotope ranges or areas is sensitive to the number of measures in a particular sample (Fig. S6). While some isotopic samples are necessarily small due to the nature of fossil preservation, the average isotopic sample size is 7.47 across species/location/time bins plotted in the main text, Fig. 4A. Accordingly, calculated values of b_{iso} are in most cases likely to be underestimating the true value. Because most species are represented by multiple location/time bins, we argue that the range of b_{iso} values of carnivore species as a function of body size is likely to be

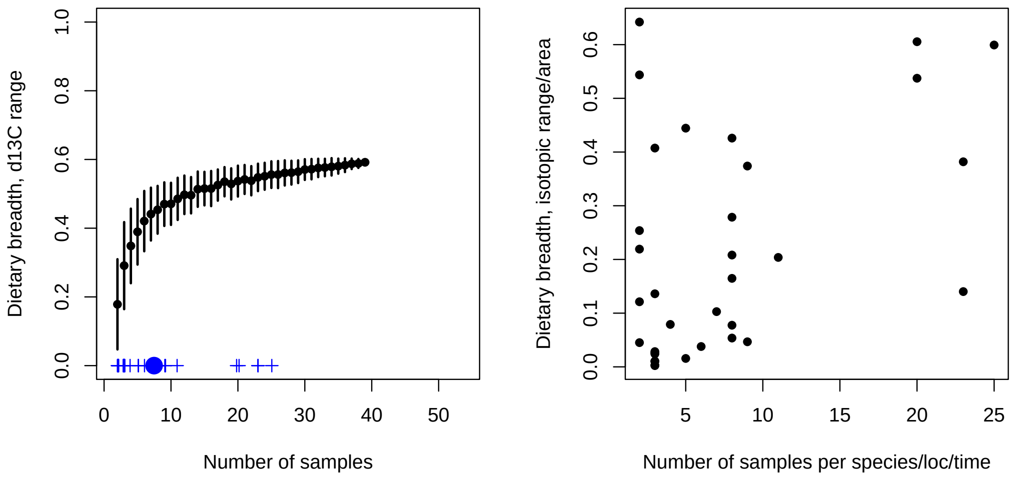

FIG. S6: (a) Estimating dietary breadth from $b_{\rm iso}$ as a function of sample size shown for contemporary hyenas, where means (black circles) and standard deviations (vertical lines) are taken across 200 replicate draws (without replacement). The blue crosses along the bottom of the plot show the number of samples per species/location/time bins used for $b_{\rm iso}$ estimates shown in the main text, Fig. 4A. The large blue circle denotes the mean sample size of 7.47. (b) There is not a strong correlation between the number of samples per species/location/time bin (main text, Fig. 4a) and dietary breadth measured from isotopic range or area ($b_{\rm iso}$; $R^2 = 0.175$, $p = 0.008$). This suggests that there is not a strong sample size bias impacting the isotopic measurement of diet breadth.

well represented in the combined dataset that we present in the main text, and it is the qualitative fit of these value ranges that we consider most relevant for comparing against model expectations.

If predators rely on the full suite of available prey (to different extents, among individuals and over time), their isotopic breadth is more likely to approximate that of the available herbivore community, such that $b_{\rm iso} \to 1$, measured from either the convex hull in (δ^{13}C, δ^{15}N) space, or from the δ^{13}C range. On the other hand, if predator individuals specialize on a particular herbivore species or set of species, it is more likely that $b_{\rm iso} < 1$. We note that whether stable carbon isotope ratios are obtained from bone collagen, enamel, or potentially other sources, our estimate of dietary breadth will not be impacted as long as measurements are obtained from the same biological source for herbivores and carnivores alike with respect

to each assemblage. We also note that spatio-temporal differences in mammalian isotope distributions will not bias our breadth metric as long as individuals representing a given assemblage are constrained to approximately the same space and time. Finally, because we compare the relative isotopic areas and ranges of predator species and herbivore communities directly, without accounting for overlap, we do not need to adjust isotopic values for trophic discrimination.

In addition to the carbon isotope values for Pleistocene carnivores and their associated herbivores assembled from published data, we include five heretofore unpublished predator species from Friesenhahn Cave (Texas), dating to the the Full Glacial, including *Homotherium*, *Smilodon floridanus*, *Ursus americanus*, and *Canis latrans*. A single specimen of *Aenocyon dirus* is also included in the dataset but not the analysis, given an isotopic range cannot be calculated from a single datum. The analytical methods used to generate isotopic data from these samples is described in Koch *et al.* (2004) and Yann *et al.* (2016).

Section S6. Duration hypercarnivores across the Cenozoic

We collated first and last appearances of terrestrial Carnivora taxa from North America spanning the Cenozoic from the Paleobiology Database (PBDB, `http://paleobiodb.org/`), with a focus on mid-late Eocene to the present. Because there are clear relationships between the dental traits of contemporary carnivorans and hypercarnivory, we focus on these species, to the exclusion of other predatory eutherian mammals such as creodonts (including oxaenidonts and hyaenodonts), entelodonts (which include *Andrewsarchus* and other large predators such as *Daeodon*), and mesonychians among others. Genera were classified as either hypercarnivorous or non-hypercarnivorous based on craniodental form, which affects skull dimensions and overall configuration (Van Valkenburgh 1991, 1999, 2007, Van Valkenburgh *et al.* 2004, Wang 1994), body size and expected prey size (Balisi & Van Valkenburgh 2020), and isotopic profile (Chatters *et al.* 2024), and include: *Aelurodon*, *Arctodus*, *Borophagus*, *Cephalogale*, *Chasmaporthetes*, *Cynodesmus*, *Daphoenictis*, *Dinictis*, *Ectopcynus*, *Enhydrocyon*, *Epicyon*, *Euoplocyon*, *Eusmilus*, *Homotherium*, *Lynx*, *Mammacyon*, *Megalictis*, *Mesocyon*, *Myracinonyix*, *Nimravides*, *Panthera*, *Paraenhydrocyon*, *Paratomarctus*, *Phoberocyon*, *Protepicyon*, *Puma*, *Smilodon*, and *Temnocyon.*. Body size estimates were primarily obtained using the CarniFOSS database (Faurby *et al.* 2021). Fossil occurrences identified

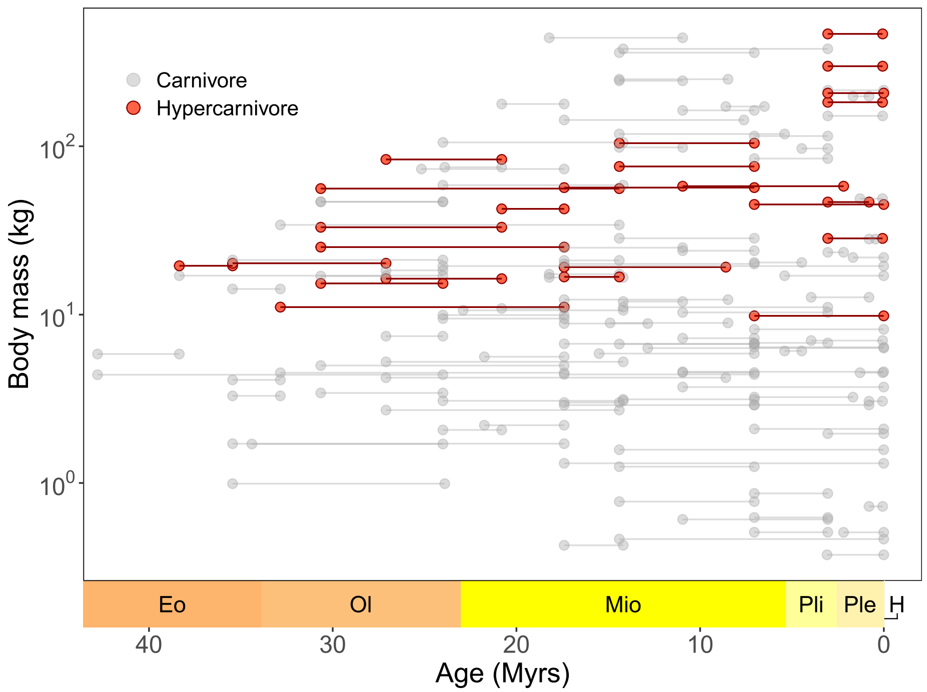

FIG. S7: Temporal occurrence of Carnivore genera from the mid-late Eocene to the present as a function of body mass (kg). Gray points and spans denote non-hypercarnivore temporal ranges; red points and spans denote hypercarnivore temporal ranges. See supplementary text for associated data sources.

only to the genus level were given the average of body mass of species from that genus. For species and genera where body mass was not found within the main source, we either utilized averages of other related genera of the same size (e.g., *Neogale* with *Neovision* and *Mustela*, *Suparictis* with *Parictis* and *Nothocyon*, *Chamitataxus* with *Taxidea*) or obtained the body mass from the literature (e.g., *Eusmilus* genus from Barrett (2021), *Protarctos abstrusus* from Wang *et al.* (2017)). We removed fossil occurrences of species for which we could not find body size estimates or relationships in the literature. Duration was calculated as last occurrence minus first occurrence (in units of Myrs). As a side note, this database results in a geometric mean body mass estimate of 79 kg for carnivore genera $>$ 20 kg, as compared to the 84 kg value calculated from Cenozoic carnivore species collated from Smith *et al.* (2003) used in the main text (see main text, Fig. 3B). The slightly higher value for the latter is likely due to the species versus genus-level resolutions used in the respective estimates.

First, we observe that large-bodied and mega-sized carnivores are a common presence in

terrestrial ecosystems throughout the assessed record, despite biases such as the pull of the recent and the lower abundance of larger-bodied apex predators (red temporal spans in Fig. S7). Second, while there are a relatively smaller number of genera within Carnivora to enter the mega-size range, many of these are non-hypercarnivores, including omnivorous ursids (e.g., *Agriotherium*, *Ursus*), and bear dogs (e.g., *Amphicyon*, *Ischyrocyon*, *Pseudocyon*). Though we do not include omnivory in our model framework, we observe subsidization (such as that from primary production obtained by omnivores) to promote feasible predator populations across the observed body size range (see discussion associated with main text, Fig. 2).

Of more interest here is the size-specific duration of hypercarnivores. Our model predicts that large-bodied non-selective hypercarnivores with an increased dietary breadth (incorporating a larger range of prey body sizes) are expected to have a population-level advantage – relative to selective predators – at size classes up to 421 kg to 668 kg. Above this size range the relative advantage of non-selective diets declines, at which point selective megapredators with low dietary breadth instead assume the advantage (see main text, Fig. 4). Because megaherbivores have lower population densities and are more vulnerable to external sources of mortality (Rallings *et al.* 2024), the intense predation pressure resulting from selective megapredators is more likely to lead to population collapse of prey, which then destabilizes that of the predator.

As discussed in the main text, these trophic dynamics may contribute to a ceiling on megapredator body size, triggered by their impacts on megaherbivore prey. Such a self-imposed limit may leave tell-tale signals in the fossil record, such as shorter duration times for megapredator size classes. Here we show that Cenozoic hypercarnivore duration, while variable across genera, is maximized at $M_p = 56$ kg (19.44 Myrs), and declines sharply above 100 kg (Fig. S8). For size classes above 100 kg, duration is maximized at 4.7 Myrs. By comparison, we do not see a similar decline in duration among mega-sized omnivores. These macroevolutionary patterns align with the notion that top-down dynamics may place energetic boundaries on body size, contributing to the selective forces driving the evolution of mammals across the Cenozoic.

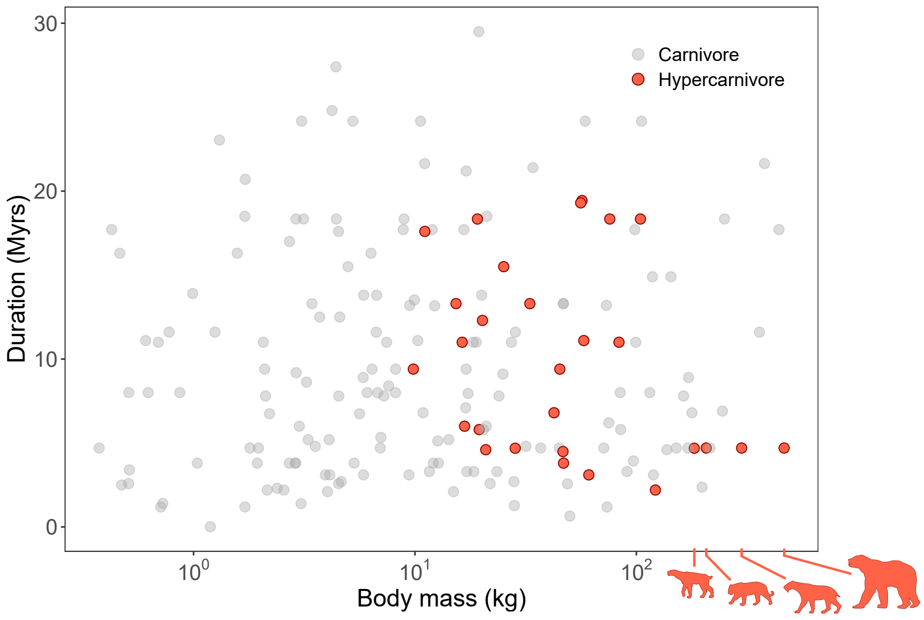

FIG. S8: Duration of Carnivora genera (Myrs) as a function of body size (kg). Gray points denote non-hypercarnivore duration; red points denote hypercarnivore duration. The four largest hypercarnivores genera included in this dataset are depicted as silhouettes, and include (from left to right) *Homotherium* (scimitar-toothed cats), *Panthera* (averaged across contemporary and extinct species, including the American lion), *Smilodon* (saber-toothed cats), and *Arctodus* (short-faced bears). *Homotherium serum* silhouette by Dantheman9758 (vectorized by T. Michael Keesey) on Phylopic.org; used with no changes under a CC Attribution 3.0 Unported license (https://creativecommons.org/licenses/by/3.0/). *Smilodon* silhouette by Ivan Iofrida on Phylopic.org; used with no changes under a CC Attribution 4.0 International deed (https://creativecommons.org/licenses/by/4.0/). *Panthera* and *Arctodus* silhouettes are from Phylopic.org and are in the public domain under a CC0 1.0 Universal Public Domain Dedication license.

TABLE S2: Carnivore species shown in Fig. 4; see online supplemental data (`mammalian_isotopes_all.xlsx`) for details. C = canids, U = ursids, H = hyaenids, F = felids.

Location	Age	Habitat	Carnivore species	Mass (kg)	*n*	Family	Source
Spain	early Pleistocene	C3	*Pachycrocuta brevirostris*	110.00	3	H	(Palmqvist *et al.* 2008)
			Homotherium latidens	183.00	3	F	(Palmqvist *et al.* 2008)
			Megantereon whitei	130.00	2	F	(Palmqvist *et al.* 2008)
			Ursus etruscus	89.10	3	U	(Palmqvist *et al.* 2008)
			Lycaon lycaonoides	30.00	3	C	(Palmqvist *et al.* 2008)
			Canis mosbachensis	22.07	3	C	(Palmqvist *et al.* 2008)
Beringia	Pre-glacial	C3	*Arctodus*	785.00	3	U	(Fox-Dobbs *et al.* 2008)
			Canis	51.50	23	C	(Fox-Dobbs *et al.* 2008)
			Homotherium	183.00	11	F	(Fox-Dobbs *et al.* 2008)
			Ursus	270.00	6	U	(Fox-Dobbs *et al.* 2008)
Beringia	Full-glacial	C3	*Canis*	51.50	7	C	(Fox-Dobbs *et al.* 2008)
			Panthera atrox	382.00	3	F	(Fox-Dobbs *et al.* 2008)
			Ursus	270.00	2	U	(Fox-Dobbs *et al.* 2008)
Beringia	Post-glacial	C3	*Canis*	51.50	9	C	(Fox-Dobbs *et al.* 2008)
			Panthera atrox	382.00	5	F	(Fox-Dobbs *et al.* 2008)
			Ursus	270.00	8	U	(Fox-Dobbs *et al.* 2008)
Friesenhahn Cave	Full-glacial	C3/C4	*Homotherium*	183.00	8	F	(DeSantis *et al.* 2021)
			Homotherium	183.00	4	F	This study
			Smilodon floridanus	220.00	2	F	This study
			Ursus americanus	298.50	2	U	This study
			Canis latrans	14.00	2	C	This study
Ingleside	Pre-glacial	C3/C4	*Aenocyon dirus*	64.00	3	C	This study
Fairmead	mid Pleistocene	C3/C4	*Aenocyon dirus*	64.00	8	C	(Trayler *et al.* 2015)
			Canis latrans	14.00	5	C	(Trayler *et al.* 2015)
			Smilodon	220.00	3	F	(Trayler *et al.* 2015)
Irvington	early Pleistocene	C3/C4	*Aenocyon dirus*	64.00	2	C	(Trayler *et al.* 2015)
La Brea	Pre-glacial	C3	*Aenocyon dirus*	64.00	8	C	(Fuller *et al.* 2014)
			Smilodon fatalis	351.00	8	F	(Fuller *et al.* 2014)
La Brea	Pre/Full-glacial	C3	*Aenocyon dirus*	64.00	25	C	(Coltrain *et al.* 2004)
			Canis latrans	14.00	9	C	(Coltrain *et al.* 2004)
			Panthera leo atrox	382.00	8	F	(Coltrain *et al.* 2004)
			Smilodon fatalis	351.00	23	F	(Coltrain *et al.* 2004)
Florida	Pleistocene	C3/C4	*Canis edwardii*	30.00	20	C	(Feranec & DeSantis 2014)
			Smilodon gracilis	77.50	20	F	(Feranec & DeSantis 2014)

Anyonge, W. & Roman, C. (2006). New body mass estimates for *Canis dirus*, the extinct Pleistocene dire wolf. *Journal of Vertebrate Paleontology*, 26, 209–212.

Balisi, M.A. & Van Valkenburgh, B. (2020). Iterative evolution of large-bodied hypercarnivory in canids benefits species but not clades. *Communications Biology*, 3, 461.

Barrett, P.Z. (2021). The largest hoplophonine and a complex new hypothesis of nimravid evolution. *Scientific Reports*, 11, 21078.

Blueweiss, L., Fox, H., Kudzma, V., Nakashima, D., Peters, R. & Sams, S. (1978). Relationships between body size and some life history parameters. *Oecologia*, 37, 257–272.

Calder III, W.A. (1983). An allometric approach to population cycles of mammals. *Journal of Theoretical Biology*, 100, 275–282.

Carbone, C. & Gittleman, J.L. (2002). A common rule for the scaling of carnivore density. *Science*, 295, 2273–2276.

Carbone, C., Mace, G.M., Roberts, S.C. & Macdonald, D.W. (1999). Energetic constraints on the diet of terrestrial carnivores. *Nature*, 402, 286–288.

Chatters, J.C., Potter, B.A., Fiedel, S.J., Morrow, J.E., Jass, C.N. & Wooller, M.J. (2024). Mammoth featured heavily in Western Clovis diet. *Science Advances*, 10, eadr3814.

Christiansen, P. & Harris, J.M. (2005). Body size of *Smilodon* (mammalia: Felidae). *Journal of Morphology*, 266, 369–384.

Coltrain, J.B., Harris, J.M., Cerling, T.E., Ehleringer, J.R., Dearing, M.D., Ward, J. & Allen, J. (2004). Rancho La Brea stable isotope biogeochemistry and its implications for the palaeoecology of late Pleistocene, coastal southern California. *Palaeogeography, Palaeoclimatology, Palaeoecology*, 205, 199–219.

Creel, S. & Creel, N.M. (2002). *The African wild dog: behavior, ecology, and conservation.* vol. 25. Princeton University Press.

Damuth, J. (1987). Interspecific allometry of population density in mammals and other animals: The independence of body mass and population energy-use. *Biological Journal of the Linnean Society*, 31, 193–246.

Dantas, M.A. (2022). Estimating the body mass of the late pleistocene megafauna from the south america intertropical region and a new regression to estimate the body mass of extinct xenarthrans.

Journal of South American Earth Sciences, 119, 103900.

DeSantis, L.R., Feranec, R.S., Antón, M. & Lundelius, E.L. (2021). Dietary ecology of the scimitar-toothed cat *Homotherium serum*. *Current Biology*, 31, 2674–2681.

Dunbrack, R.L. & Ramsay, M.A. (1993). The allometry of mammalian adaptations to seasonal environments: A critique of the fasting endurance hypothesis. *Oikos*, 66, 336.

Faurby, S., Morlo, M. & Werdelin, L. (2021). Carnifoss: A database of the body mass of fossil carnivores. *Global Ecology and Biogeography*, 30, 1958–1964.

Feranec, R.S. & DeSantis, L.R. (2014). Understanding specifics in generalist diets of carnivorans by analyzing stable carbon isotope values in Pleistocene mammals of Florida. *Paleobiology*, 40, 477–493.

Figueirido, B., Pérez-Claros, J.A., Hunt, R.M. & Palmqvist, P. (2011). Body mass estimation in amphicyonid carnivoran mammals: a multiple regression approach from the skull and skeleton. *Acta Palaeontologica Polonica*, 56, 225–246.

Flower, L. (2016). New body mass estimates of british pleistocene wolves: Palaeoenvironmental implications and competitive interactions. *Quaternary Science Reviews*, 149, 230–247.

Fox-Dobbs, K., Leonard, J.A. & Koch, P.L. (2008). Pleistocene megafauna from eastern beringia: Paleoecological and paleoenvironmental interpretations of stable carbon and nitrogen isotope and radiocarbon records. *Palaeogeography, Palaeoclimatology, Palaeoecology*, 261, 30–46.

Fuller, B.T., Fahrni, S.M., Harris, J.M., Farrell, A.B., Coltrain, J.B., Gerhart, L.M., Ward, J.K., Taylor, R. & Southon, J.R. (2014). Ultrafiltration for asphalt removal from bone collagen for radiocarbon dating and isotopic analysis of Pleistocene fauna at the tar pits of Rancho La Brea, Los Angeles, California. *Quaternary Geochronology*, 22, 85–98.

Fuller, B.T., Southon, J.R., Fahrni, S.M., Farrell, A.B., Takeuchi, G.T., Nehlich, O., Guiry, E.J., Richards, M.P., Lindsey, E.L. & Harris, J.M. (2020). Pleistocene paleoecology and feeding behavior of terrestrial vertebrates recorded in a pre-LGM asphaltic deposit at Rancho La Brea, California. *Palaeogeography, Palaeoclimatology, Palaeoecology*, 537, 109383.

Gazin, C.L. (1942). The late cenozoic vertebrate faunas from the san pedro valley, ariz. *Proceedings of the United States National Museum*.

Hayward, M. (2006). Prey preferences of the spotted hyaena (Crocuta crocuta) and degree of dietary overlap with the lion (Panthera leo). *Journal of Zoology*, 270, 606–614.

Hayward, M., Henschel, P., O'Brien, J., Hofmeyr, M., Balme, G. & Kerley, G.I. (2006a). Prey

preferences of the leopard (*Panthera pardus*). *Journal of Zoology*, 270, 298–313.

Hayward, M., Hofmeyr, M., O'brien, J. & Kerley, G.I. (2006b). Prey preferences of the cheetah (*Acinonyx jubatus*)(Felidae: Carnivora): Morphological limitations or the need to capture rapidly consumable prey before kleptoparasites arrive? *Journal of Zoology*, 270, 615–627.

Hayward, M.W. & Kerley, G. (2008). Prey preferences and dietary overlap amongst Africa's large predators. *South African Journal of Wildlife Research*, 38, 93–108.

Hayward, M.W. & Kerley, G.I. (2005). Prey preferences of the lion (*Panthera leo*). *Journal of Zoology*, 267, 309–322.

Hayward, M.W., O'Brien, J., Hofmeyr, M. & Kerley, G.I. (2006c). Prey preferences of the African wild dog *Lycaon pictus* (Canidae: Carnivora): Ecological requirements for conservation. *Journal of Mammalogy*, 87, 1122–1131.

Hill, M.G. & Easterla, D.A. (2023). A complete sabertooth cat cranium from the midcontinent of north america and its evolutionary and ecological context. *Quaternary Science Reviews*, 307, 108045.

Hou, C., Zuo, W., Moses, M.E., Woodruff, W.H., Brown, J.H. & West, G.B. (2008). Energy uptake and allocation during ontogeny. *Science*, 322, 736–739.

Jackson, A.L., Inger, R., Parnell, A.C. & Bearhop, S. (2011). Comparing isotopic niche widths among and within communities: Siber–stable isotope bayesian ellipses in r. *Journal of Animal Ecology*, 80, 595–602.

Kempes, C.P., Dutkiewicz, S. & Follows, M.J. (2012). Growth, metabolic partitioning, and the size of microorganisms. *Proceedings of the National Academy of Sciences U.S.A.*, 109, 495–500.

Koch, P.L., Diffenbaugh, N.S. & Hoppe, K.A. (2004). The effects of late Quaternary climate and pCO_2 change on C_4 plant abundance in the south-central United States. *Palaeogeography, Palaeoclimatology, Palaeoecology*, 207, 331–357.

Kondoh, M. (2003). Foraging Adaptation and the Relationship Between Food-Web Complexity and Stability. *Science*, 299, 1388–1391.

Koufos, G.D., Konidaris, G.E. & Harvati, K. (2018). Revisiting ursus etruscus (carnivora, mammalia) from the early pleistocene of greece with description of new material. *Quaternary International*, 497, 222–239.

Marciszak, A. & Lipecki, G. (2022). Panthera gombaszoegensis (kretzoi, 1938) from poland in the scope of the species evolution. *Quaternary International*, 633, 36–51.

Merrill, A. & Watt, B. (1973). Part 2: digestibility and available energy of foods. *Energy value of foods: basis and derivation. Agriculture handbook No*, 74, 8–24.

Moses, M.E., Hou, C., Woodruff, W.H., West, G.B., Nekola, J.C., Zuo, W. & Brown, J.H. (2008). Revisiting a Model of Ontogenetic Growth: Estimating Model Parameters from Theory and Data. *http://dx.doi.org.proxy.lib.sfu.ca/10.1086/679735*, 171, 632–645.

Palmqvist, P., Martínez-Navarro, B. & Arribas, A. (1996). Prey selection by terrestrial carnivores in a lower pleistocene paleocommunity. *Paleobiology*, 22, 514–534.

Palmqvist, P., Mendoza, M., Arribas, A. & Gröcke, D.R. (2002). Estimating the body mass of pleistocene canids: discussion of some methodological problems and a new 'taxon free'approach. *Lethaia*, 35, 358–360.

Palmqvist, P., Pérez-Claros, J.A., Janis, C.M. & Gröcke, D.R. (2008). Tracing the ecophysiology of ungulates and predator–prey relationships in an early Pleistocene large mammal community. *Palaeogeography, Palaeoclimatology, Palaeoecology*, 266, 95–111.

Pawar, S., Dell, A.I. & Savage, V.M. (2012). Dimensionality of consumer search space drives trophic interaction strengths. *Nature*.

Pirt, S. (1965). The maintenance energy of bacteria in growing cultures. *Proc. Roy. Soc. B*, 163, 224.

Prange, H.D., Anderson, J.F. & Rahn, H. (1979). Scaling of skeletal mass to body mass in birds and mammals. *The American Naturalist*, 113, 103–122.

Rallings, T., Kempes, C.P. & Yeakel, J.D. (2024). On the dynamics of mortality and the ephemeral nature of mammalian megafauna. *The American Naturalist*, 204, 274–288.

Savage, V.M., Gillooly, J.F., Brown, J.H., West, G.B. & Charnov, E.L. (2004). Effects of Body Size and Temperature on Population Growth. *http://dx.doi.org.proxy.lib.sfu.ca/10.1086/679735*, 163, 429–441.

Scheel, D. (1993). Profitability, encounter rates, and prey choice of african lions. *Behavioral ecology*, 4, 90–97.

Sherani, S. (2016). A new specimen-dependent method of estimating felid body mass. *PeerJ Preprints*, 4, e2327v1.

Sinclair, A.R.E., Mduma, S. & Brashares, J.S. (2003). Patterns of predation in a diverse predator–prey system. *Nature*, 425, 288–290.

Smith, F., Lyons, S., Ernest, S., Jones, K., Kaufman, D., Dayan, T., Marquet, P.A., Brown, J.H.

& Haskell, J. (2003). Body mass of late Quaternary mammals. *Ecology*, 84, 3403–3403.

Sorkin, B. (2006). Ecomorphology of the giant bear-dogs *Amphicyon* and *Ischyrocyon*. *Historical Biology*, 18, 375–388.

Stryer, L. (1995). *Biochemistry, Fourth Edition*. W.H. Freeman and Company, Newyork, NY.

Trayler, R.B., Dundas, R.G., Fox-Dobbs, K. & Van De Water, P.K. (2015). Inland California during the Pleistocene—Megafaunal stable isotope records reveal new paleoecological and paleoenvironmental insights. *Palaeogeography, Palaeoclimatology, Palaeoecology*, 437, 132–140.

Uiterwaal, S.F., Lagerstrom, I.T., Lyon, S.R. & DeLong, J.P. (2018). Data paper: Forage (functional responses from around the globe in all ecosystems) database: a compilation of functional responses for consumers and parasitoids. *BioRxiv*, p. 503334.

Valdovinos, F.S., Hale, K.R., Dritz, S., Glaum, P.R., McCann, K.S., Simon, S.M., Thébault, E., Wetzel, W.C., Wootton, K.L. & Yeakel, J.D. (2023). A bioenergetic framework for aboveground terrestrial food webs. *Trends in Ecology & Evolution*, 38, 301–312.

Valdovinos, F.S., Ramos-Jiliberto, R., Garay-Narváez, L., Urbani, P. & Dunne, J.A. (2010). Consequences of adaptive behaviour for the structure and dynamics of food webs. *Ecology Letters*, 13, 1546–1559.

Van Valkenburgh, B. (1991). Iterative evolution of hypercarnivory in canids (mammalia: Carnivora): evolutionary interactions among sympatric predators. *Paleobiology*, 17, 340–362.

Van Valkenburgh, B. (1999). Major patterns in the history of carnivorous mammals. *Annual Review of Earth and Planetary Sciences*, 27, 463–493.

Van Valkenburgh, B. (2007). Déjà vu: the evolution of feeding morphologies in the carnivora. *Integrative and comparative biology*, 47, 147–163.

Van Valkenburgh, B., Wang, X. & Damuth, J. (2004). Cope's rule, hypercarnivory, and extinction in North American canids. *Science*, 306, 101–104.

Wang, X. (1994). Phylogenetic systematics of the hesperocyoninae (carnivora: Canidae). *Bulletin of the American Museum of Natural History*, pp. 1–207.

Wang, X., Rybczynski, N., Harington, C.R., White, S.C. & Tedford, R.H. (2017). A basal ursine bear (protarctos abstrusus) from the pliocene high arctic reveals eurasian affinities and a diet rich in fermentable sugars. *Scientific Reports*, 7, 17722.

West, G.B., Brown, J.H. & Enquist, B.J. (1997). A general model for the origin of allometric scaling laws in biology. *Science*, 276, 122–126.

West, G.B., Brown, J.H. & Enquist, B.J. (2001). A general model for ontogenetic growth. *Nature*, 413, 628–631.

Yann, L.T., DeSantis, L.R., Koch, P.L. & Lundelius, E.L. (2016). Dietary ecology of Pleistocene camelids: influences of climate, environment, and sympatric taxa. *Palaeogeography, Palaeoclimatology, Palaeoecology*, 461, 389–400.

Yeakel, J.D., Hutchinson, M.C., Kempes, C.P., Koch, P.L., Ugarte, P.D.S., Gill, J.L. & Pires, M.M. (2025). Supplementary electronic data for "Bioenergetic trophic trade-offs determine mass-dependent extinction thresholds across the Cenozoic". https://doi.org/10.5281/zenodo.18842091.

Yeakel, J.D., Kempes, C.P. & Redner, S. (2018). Dynamics of starvation and recovery predict extinction risk and both Damuth's law and Cope's rule. *Nature Communications*, 9, 1–10.
